# Terrestrial planet formation from lost inner solar system material

**DOI:** 10.1126/sciadv.abj7601

**Published:** 2021-12-22

**Authors:** Christoph Burkhardt, Fridolin Spitzer, Alessandro Morbidelli, Gerrit Budde, Jan H. Render, Thomas S. Kruijer, Thorsten Kleine

**Affiliations:** 1 Institut für Planetologie, University of Münster, Wilhelm-Klemm-Str. 10, 48149 Münster, Germany.; 2 Laboratoire Lagrange, UMR7293, Université de Nice Sophia-Antipolis, CNRS, Observatoire de la Côte d’Azur, Boulevard de l’Observatoire, 06304 Nice, Cedex 4, France.; 3 Division of Geological and Planetary Sciences, California Institute of Technology, 1200 E California Blvd, Pasadena, CA 91125, USA.; 4 Museum für Naturkunde, Leibniz Institute for Evolution and Biodiversity Science, Invalidenstraße 43, 10115 Berlin, Germany.; 5 Institut für Geologische Wissenschaften, Freie Universität Berlin, Malteserstraße 74-100, 12249 Berlin, Germany.; 6 Max Planck Institute for Solar System Research, Justus-von-Liebig-Weg 3, 37077 Göttingen, Germany.

## Abstract

Two fundamentally different processes of rocky planet formation exist, but it is unclear which one built the terrestrial planets of the solar system. They formed either by collisions among planetary embryos from the inner solar system or by accreting sunward-drifting millimeter-sized “pebbles” from the outer solar system. We show that the isotopic compositions of Earth and Mars are governed by two-component mixing among inner solar system materials, including material from the innermost disk unsampled by meteorites, whereas the contribution of outer solar system material is limited to a few percent by mass. This refutes a pebble accretion origin of the terrestrial planets but is consistent with collisional growth from inner solar system embryos. The low fraction of outer solar system material in Earth and Mars indicates the presence of a persistent dust-drift barrier in the disk, highlighting the specific pathway of rocky planet formation in the solar system.

## Teaser

An integrated assessment of isotopic variations among meteorites reveals the process by which Earth and Mars are formed.

## INTRODUCTION

**Fig. 1.**
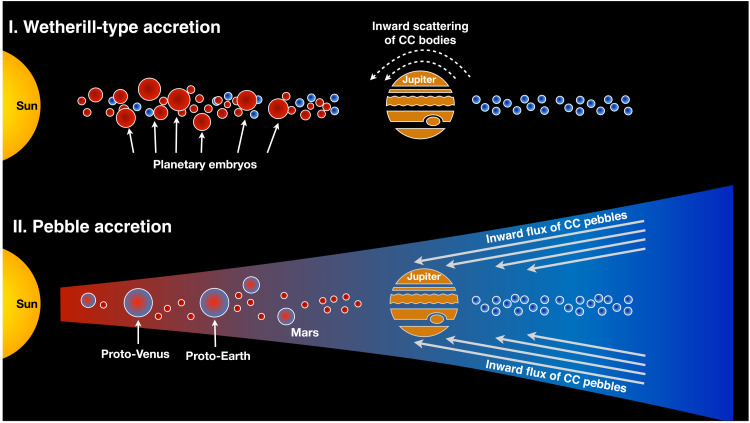
Possible scenarios of terrestrial planet formation. In the classic “Wetherill-type” model of oligarchic growth, the terrestrial planets formed by mutual collisions among Moon- to Mars-sized planetary embryos after the gas disk dissipated and accreted only a small fraction of CC planetesimals, which were scattered inward during Jupiter’s growth and/or putative migration. Alternatively, the terrestrial planets may have formed within the lifetime of the gas disk by efficiently accreting “pebbles” from the outer solar system, which drift sunward through the disk due to gas drag. The two models differ in the amount of outer solar system (CC) material accreted by the terrestrial planets, which may be quantified using nucleosynthetic isotope anomalies.

Rocky planets may have formed by two fundamentally different processes (Fig. 1). In the classic model of oligarchic growth, the accretion of Moon- to Mars-sized planetary embryos in the protoplanetary disk of gas and planetesimals was followed, after gas removal, by a protracted phase of mutual impacts among the embryos, leading to the final assembly of the terrestrial planets (*1*). More recently, an alternative model has been proposed, in which planets grow by accreting “pebbles” from the outer solar system, which drift sunward through the disk because of gas drag (*2*, *3*). Pebble accretion is very effective at forming giant planet cores (*4*, *5*) and may have also formed the terrestrial planets of the solar system (*6*, *7*). Determining which of these two processes governed the formation of the terrestrial planets of the solar system is crucial for understanding the solar system’s architecture and dynamical evolution and for placing planet formation in the solar system into the context of general planet formation processes, such as those observed in exoplanetary systems.

Oligarchic growth and pebble accretion are both dynamically possible, and so, they cannot be distinguished solely on theoretical grounds. Instead, the answer may be sought in the origin of the accreted material, because the two accretion models predict vastly different amounts of outer solar system material incorporated into the terrestrial planets. In the oligarchic growth model, the terrestrial planets predominantly accreted from embryos from the inner solar system, with only a few percentage of a planet’s mass coming from outer solar system bodies (*8*). By contrast, in the pebble accretion model, much of the terrestrial planets accreted from dust and pebbles drifting inward from the outer solar system, amounting to ~30 to 50% by mass of outer solar system material in Earth and Mars (*7*, *9*, *10*). Thus, the amount of outer solar system material accreted by the terrestrial planets is the key discriminant between the oligarchic growth and pebble accretion models (Fig. 1).

The amount of outer solar system material accreted by the terrestrial planets may be determined using nucleosynthetic isotope anomalies. These arise through the heterogeneous distribution of presolar matter within the solar protoplanetary disk and provide a record of the heritage of a planet’s building material (*11*–*14*). In particular, these isotope anomalies can distinguish between noncarbonaceous (NC) and carbonaceous (CC) meteorites, which most likely represent the inner and outer solar system, respectively (*11*). Thus, the isotopic compositions of Earth and Mars with respect to the NC and CC reservoirs provide a pathway for determining how much outer solar system material was accreted by these bodies. However, whereas some studies concluded that Earth and Mars largely (>95%) accreted from inner solar system (NC) material (*13*, *15*, *16*), which would be consistent with the oligarchic growth model, others argued that Earth and Mars accreted substantial amounts (~30 to 50%) of CC material (*9*, *10*), which in turn would be consistent with a pebble accretion origin (*7*). Hence, current interpretations of the isotopic data cannot distinguish between the two models of terrestrial planet formation, because both low and high amounts of outer solar system material in the terrestrial planets have been inferred.

These disparate conclusions reflect that previous studies either considered isotope anomalies of only a subset of elements (*9*, *10*) or made specific assumptions about which combination of known meteorites best represents the precursor material of the planets (*13*). Moreover, these studies neglected the observation that Earth likely incorporated material that remained unsampled among meteorites (*12*, *17*–*19*), in which case the amount of CC material in Earth cannot be determined by assuming that meteorites represent its building material. Consequently, accurately determining the bulk fraction of outer solar system material in the terrestrial planets in a self-consistent manner requires not only that the isotope anomalies of a representative set of elements are considered but also an assessment of the full isotopic variability among the precursor objects of the terrestrial planets, including the planetary building material that remained unsampled among meteorites.

Here, we use the recent observation of correlated isotope variations among NC meteorites (*20*) to show that both Earth and Mars incorporated material unsampled among meteorites, determine the provenance and isotopic composition of this lost planetary building material, and use this information to assess the amount of CC material accreted by Earth and Mars. To this end, we also report new high-precision Ti, Zr, and Mo isotopic data for martian meteorites, which are crucial for better defining the isotopic composition of Mars with respect to NC and CC meteorites and, therefore, for determining the fraction of outer solar system material accreted by Mars.

## RESULTS

### Isotope composition of Mars

The isotopic compositions of Ti, Zr, and Mo in Mars are poorly defined because until now only a few samples have been analyzed, and because the precision of prior data is insufficient to unambiguously resolve the small isotopic shifts that may arise through the incorporation of CC material into Mars. To resolve these issues, we obtained Ti, Zr, and Mo isotope data for a comprehensive set of martian meteorites, including samples from the major geochemical reservoirs on Mars as sampled by meteorites (Materials and Methods). Hence, the new isotopic data of this study provide a good estimate for the isotopic composition of bulk silicate Mars (BSM) as sampled by meteorites. All isotopic data are reported in the ε-notation as parts–per–ten thousand deviations from terrestrial standard values (see Materials and Methods).

**Fig. 2.**
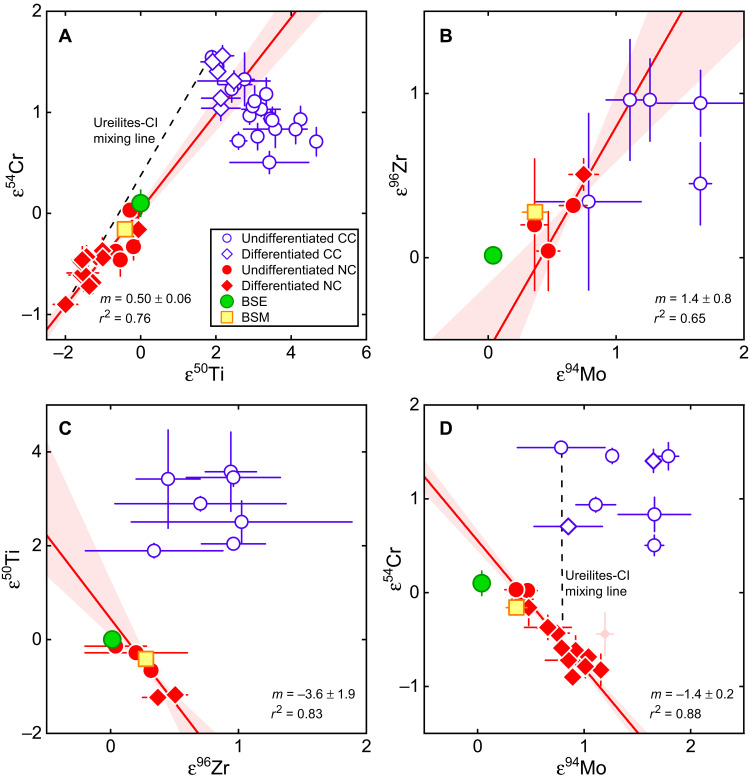
Isotope anomalies of meteorites and silicate portions of Earth (BSE) and Mars (BSM). NC meteorites representing the inner solar system are shown in red, and CC meteorites representing the outer solar system are shown in blue. For all element pairs, the isotope anomalies among NC meteorites are linearly correlated, with the BSE plotting at one end of these correlations. Solid red lines indicate linear regressions for NC data with corresponding 1σ error envelopes. (**A**) Supernova-associated isotope anomalies in ε^50^Ti and ε^54^Cr are positively correlated among NC meteorites. The BSE and BSM plot at the upper end of this correlation pointing toward CC meteorites. (**B**) Anomalies in ε^96^Zr and ε^94^Mo are also correlated among NC meteorites, but the BSE plots at the end of the NC meteorite trend pointing away from CC meteorites, toward compositions enriched in *s*-process nuclides. (**C** and **D**) BSE also plots at the end of NC correlations for anomalies in Fe-group elements (ε^50^Ti and ε^54^Cr) and heavier elements, again pointing away from CC meteorites. Dashed line in (A) indicates proposed mixing scenario between ureilite-like and CI-like materials (*9*, *10*), but this mixture cannot account for the compositions of BSE and BSM in multidimensional isotope space (D). The relationships exemplified here are valid for anomalies in all elements (Fig. 5). Brachinites [light red diamond in (D)] show decoupled Mo-Ru isotope systematics (*52*) and were excluded from the regression. Data and data sources are given in the Supplementary Materials. Error bars indicate 95% confidence intervals.

The 10 martian meteorites analyzed in this study have indistinguishable Ti isotopic compositions (table S1), with a mean ε^50^Ti anomaly of −0.42 ± 0.05 (95% confidence interval). This value is consistent with, but more precise than, the mean ε^50^Ti values calculated from martian meteorite data reported in prior studies (*21*–*23*), where some individual samples indicated scatter outside the quoted uncertainties (fig. S1). Our results corroborate earlier conclusions that the Ti isotopic composition of Mars is intermediate between enstatite and ordinary chondrites, which belong to the NC group of meteorites (Fig. 2 and fig. S1). The Zr isotope composition of Mars has so far not been precisely determined. To this end, we report high-precision Zr isotope data for six martian meteorites, which all exhibit indistinguishable Zr isotope anomalies (table S2) with a mean ε^96^Zr of 0.28 ± 0.03 (95% confidence interval) (Fig. 2 and fig. S2). As for Ti, the Zr isotopic composition for Mars is intermediate between enstatite and ordinary chondrites and also overlaps with the composition of HED (howardite-eucrite-diogenite) meteorites (fig. S2).

**Fig. 3.**
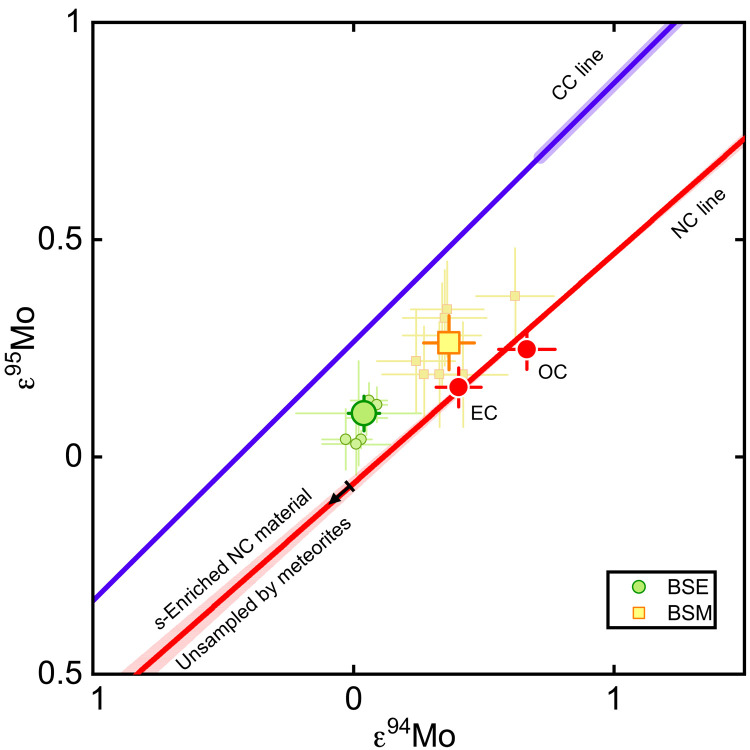
Anomalies in ε^95^Mo and ε^94^Mo for BSE and BSM. NC and CC meteorites plot on two approximately parallel lines, the NC and CC lines (*18*, *20*). The isotopic variations along these lines predominantly reflect *s*-process variations, whereas the offset between them reflects an *r*-process excess in the CC over the NC reservoir. Solid red line indicates linear regression for NC data with corresponding 1σ error envelope (*20*). Both the BSE and BSM as defined by averaging Mo isotope data (small symbols) of terrestrial mantle derived rocks (*18*) and martian meteorites plot between the lines, indicating the accretion of some CC material to both Earth and Mars. Also shown is the position of the unsampled, *s*-process–enriched material from the inner solar system (starting at arrow). The composition of the BSE and the BSM can be reproduced as a mixture between this component and known NC and CC meteorites (blue shaded area on CC line). Error bars indicate 95% confidence intervals.

We also report results of eight high-precision Mo isotope measurements on a total of ~22 g of martian material, representing 17 different meteorites (Materials and Methods and table S3). Owing to the low Mo contents of martian meteorites and the limited mass available for some of the samples, the Mo from some samples has been combined for a single Mo isotope analysis (see Material and Methods). This is justified because any isotope heterogeneity inherited from Mars’ building blocks has likely been homogenized during martian differentiation, as is evident from the homogeneous isotope composition among individual martian meteorites observed for other elements like Ti and Zr. Consistent with this, all martian meteorites analyzed in this study have identical Mo isotope anomalies within the external reproducibility (2 SD) of the measurements. In a diagram of ε^95^Mo versus ε^94^Mo, the martian meteorites plot between the NC and CC lines (Fig. 3), indicating that the BSM’s Mo derives from both the NC and CC reservoirs. Thus, unlike Ti and Zr, which reveal no unambiguous evidence for the presence of CC material in Mars, the Mo isotopic data indicate that Mars accreted some CC material. The fraction of CC-derived Mo, as calculated using the lever rule from the BSM’s position between the NC and CC lines, is 0.4 ± 0.3 (2 SD), which is similar to the value of 0.46 ± 0.15 calculated for the bulk silicate Earth (BSE) using the same approach (*18*). The calculation of CC-derived Mo mass fractions in this manner is strictly valid only if the NC and CC lines are parallel, but recent work has shown that the NC line has a slightly shallower slope than the CC line (*20*). Nevertheless, the effect of these slightly different slopes on the calculated CC-derived Mo mass fractions in the BSM and BSE is less than 0.1 and, therefore, smaller than the overall uncertainty on these mass fractions. Last, as a moderately siderophile element, Mo in BSM records only the last ~20% of its accretion (Materials and Methods), and so, the fraction of CC material accreted by Mars during its entire accretion history cannot be determined from the Mo isotopic data alone but requires considering the isotopic composition of Mars in multielement isotope space (see below).

### Building material of Earth and Mars

**Fig. 4.**
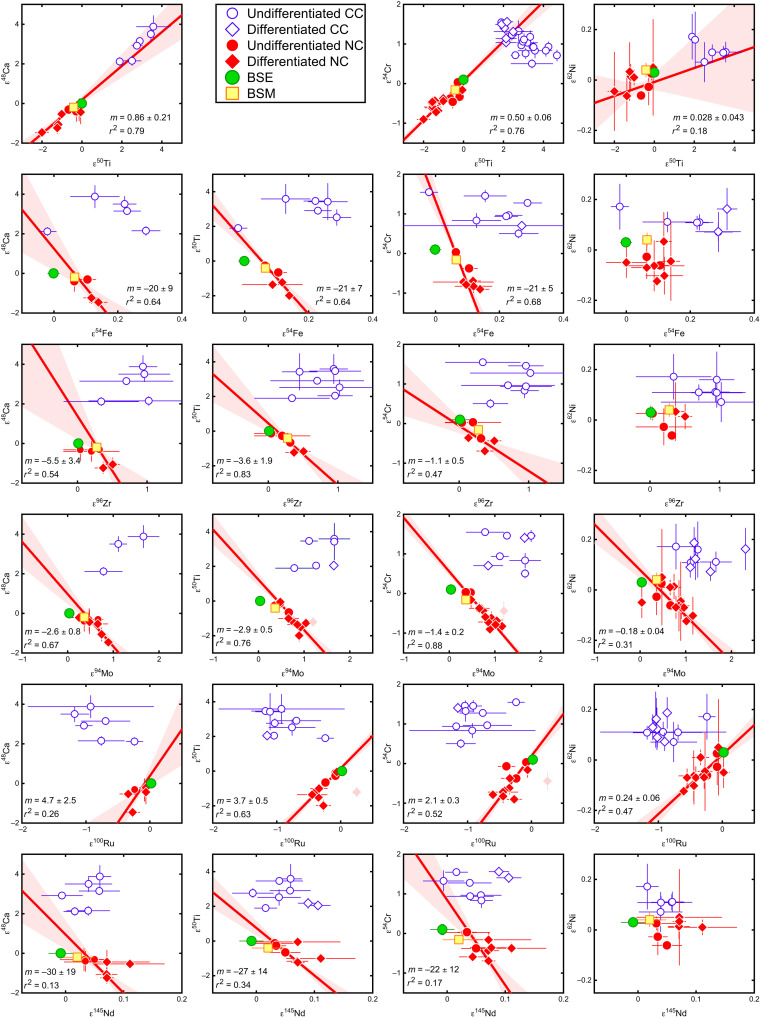
Isotope anomalies of NC and CC meteorites and silicate portions of Earth and Mars in multielement space. NC meteorites representing the inner solar system are shown in red, and CC meteorites representing the outer solar system are shown in blue. The isotope anomalies among NC meteorites are linearly correlated, with the BSE always plotting at one end of these correlations. Solid red lines indicate linear regression (York-fit) of isotopic data for NC meteorites with corresponding 1σ error envelopes. Brachinite meteorites have decoupled Mo and Ru isotope systematics (*52*) and were excluded from regressions in ε^50^Ti and ε^54^Cr versus ε^94^Mo and ε^100^Ru space (light red diamonds). Limited data, data precision, or data spread do currently not allow for meaningful NC regressions in ε^62^Ni-ε^54^Fe, ε^62^Ni-ε^96^Zr, and ε^62^Ni-ε^145^Nd space. Data are given in data S1. Uncertainties represent 95% confidence intervals.

The new isotopic data obtained in this study enable direct comparison of the isotopic compositions of BSE, BSM, and meteorites. A key observation of this comparison is that the BSE plots at one end of multielement isotope correlations defined by NC meteorites, while the BSM plots close to the compositions of enstatite and ordinary chondrites (Fig. 2). These correlations include isotope anomalies of different nucleosynthetic origin [i.e., supernova- and asymptotic giant branch (AGB) star–derived nuclides] and comprise elements with different geo- and cosmochemical behavior, indicating that the isotope variability among NC meteorites reflects mixing between two isotopically distinct components (*20*). Moreover, the NC isotope correlations reveal a notable difference between the Fe group (e.g., Ca, Ti, Cr, and Ni) and heavier elements (e.g., Zr, Mo, Ru, and Nd). For the former, the isotope anomalies are positively correlated among NC meteorites, and BSE and BSM plot at the end of this correlation pointing toward CC meteorites (Fig. 2A). Thus, for these elements, the isotopic variations among NC meteorites, Earth, and Mars are readily described as mixtures of known NC and CC materials (*10*, *11*, *13*). However, plotting the isotope anomalies of the heavier elements against each other (Fig. 2B) or against those of the Fe-group elements (Fig. 2, C and D) reveals that the BSE plots at the end of the NC meteorite trend pointing away from CC meteorites toward lower ε^94^Mo and ε^96^Zr, which represent compositions enriched in nuclides produced in the slow neutron capture process (*s*-process), which takes place in AGB stars. Note that although Earth is isotopically always close to enstatite chondrites, its isotopic composition is distinct and plots beyond the range of compositions recorded in meteorites. This corroborates earlier conclusions that Earth incorporated material that is unsampled among meteorites and, compared to known NC meteorites, is enriched in *s*-process matter (e.g., lower ε^94^Mo and ε^96^Zr) (*12*, *17*, *18*, *24*). However, the multielement isotope correlations among NC meteorites reveal that this unsampled material is also enriched in supernova-derived isotopes of some Fe-group elements (e.g., ε^50^Ti and ε^54^Cr). This observation holds for all elements showing well-resolved nucleosynthetic isotope variations among NC meteorites (Fig. 4) and, therefore, is a ubiquitous feature of the building material of the terrestrial planets in the solar system.

The observation that BSE and BSM plot on or close to the NC isotope correlation lines suggests that Earth and Mars predominantly accreted from NC material whose compositions followed the same isotopic gradient as the NC meteorites and, at least for Earth, include the unsampled *s*-process–enriched component (Fig. 2, B to D). By contrast, CC bodies from the outer solar system do not seem to have been a major contributor to Earth and Mars, because otherwise, the BSE and BSM would plot off the NC isotope trends toward the composition of the CC reservoir. Nevertheless, some CC addition to Earth and Mars is necessary to account for the observation that the BSE and BSM plot between the NC and CC lines in the ε^95^Mo-ε^94^Mo diagram (Fig. 3). To assess these observations more quantitatively, we have developed a mixing model that reduces the isotopic composition of a planet’s silicate mantle (i.e., BSE or BSM) to the contribution of three main components: (i) NC material contributing to the Mo in the planet’s present-day mantle, (ii) NC material not contributing to the Mo in the planet’s present-day mantle, and (iii) CC material (see Material and Methods). The distinction between two NC components is necessary because the mantle’s isotopic composition for a siderophile element such as Mo predominantly records the later stages of accretion (*13*, *18*). With this model, the fraction of the mantle’s Mo delivered by NC bodies and the Mo isotopic composition of these bodies can be determined from the BSE’s or BSM’s ε^94^Mo and ε^95^Mo (*18*), the ε^95^Mo-ε^94^Mo relationship among NC meteorites (*20*), and the average ε^94^Mo and ε^95^Mo of CC meteorites (*25*). As the isotopic compositions of both NC components for all other elements depend on the respective values of ε^94^Mo via the NC correlation lines, the bulk fraction of CC material in Earth and Mars can be determined by using the isotope correlations of two lithophile elements (that is, elements unaffected by core formation), ε^54^Cr (or ε^50^Ti) and ε^96^Zr, versus ε^94^Mo (Fig. 2, B and D). Last, a Monte Carlo approach was used to account for the associated uncertainties on all input quantities (e.g., the slope of the NC correlation lines or the isotopic variations among CC meteorites) and to calculate probability distribution functions (PDFs) for (i) the fraction of the mantle’s Mo delivered by NC bodies, (ii) the Mo isotopic composition of these bodies, and (iii) the bulk CC fraction accreted by Earth and Mars (see Material and Methods).

**Fig. 5.**
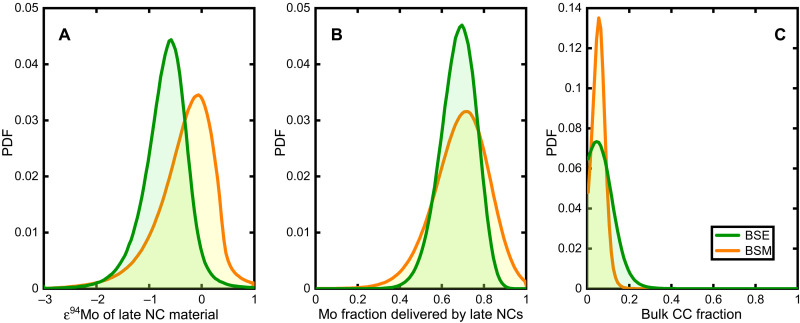
Results of Monte Carlo simulations for reproducing the isotopic composition of Earth and Mars in multidimensional isotope space. (**A**) PDF of average ε^94^Mo delivering NC Mo to the BSE and BSM. (**B**) PDF of fraction of BSE and BSM Mo delivered by NC (the complement being from CC). (**C**) PDF of fraction of bulk Earth and Mars delivered by CC material, with a peak at 4% for both planets.

The model reveals that about 70% of the BSE’s Mo was delivered by NC material with an average isotopic composition peaking at an ε^94^Mo value of about −0.6 (Fig. 5, A and B), indicating that most of the BSE’s Mo derives from NC objects that, on average, are distinct from known meteorites, all of which are characterized by positive ε^94^Mo. This does not mean that all NC material accreted by Earth had this particular ε^94^Mo, but rather that the NC accretionary mix probably included objects with more positive (such as NC meteorites) and more negative ε^94^Mo (the unsampled material from above). The offset of the BSE from the ε^94^Mo-ε^54^Cr correlation line (Fig. 2D) indicates that later accreted objects had a composition further up on the correlation line than earlier accreted objects. This is because a siderophile element such as Mo is strongly depleted in proto-Earth’s mantle due to core formation, and so for siderophile-lithophile element pairs, mixing lines between two isotopically distinct impactors are curved. Late impactors, therefore, had a stronger effect on the BSE’s Mo than on its Cr isotopic composition, and so, the offset of the BSE to the left of the ε^94^Mo-ε^54^Cr correlation line implies that late impactors had a composition further up on the line.

The results for Mars are very similar to those for Earth, but the average ε^94^Mo of the NC material that contributed to the BSM’s Mo is shifted toward slightly more positive values peaking at a value of ~0 (Fig. 5A). Moreover, unlike the BSE, Mars is not clearly offset from the ε^94^Mo-ε^54^Cr correlation line (Fig. 2D).

A key result of our model is that we infer only a small fraction of CC material (with a peak at ~4%) for Earth and Mars (Fig. 5C). Note that the larger CC fractions of ~30 to 40% inferred by considering solely the Mo isotopic data are not representative for the bulk fraction of CC material in Earth and Mars, but only refer to the last ~10 to 20% of accretion (see above). Instead, the bulk CC fraction in both planets can only be determined by considering isotopic data for all elements together, including lithophile elements. Our finding that Earth and Mars incorporated only a small bulk fraction of CC material has far-reaching implications for understanding the fundamental process of terrestrial planet formation in the solar system.

## DISCUSSION

### Lost planetary building material from the inner solar system

The low mass fraction (~4%) of outer solar system material in Earth and Mars determined in this study is in stark contrast to prior proposals that the isotopic composition of Earth and Mars reflect ~1:1 and ~ 2:1 mixtures of inner disk material having an ureilite-like isotopic composition with inward-drifting CI-chondrite–like dust from the outer solar system (*9*, *10*). This large fraction of CC material was inferred solely on the basis of isotope anomalies in Fe-group elements for which, as noted above, BSE and BSM plot at the end of the NC isotope correlation that points toward the CC compositional field (Fig. 2A). However, in multielement isotope space, mixtures between ureilites and CI chondrites do not pass through the compositions of BSE and BSM, which instead plot furthest away from these mixing lines (Fig. 2, C and D). Thus, the accretion of large amounts of CC material to Earth and Mars would only be possible if this material was enriched in *s*-process matter (i.e., negative ε^94^Mo and ε^96^Zr) and, in this case, would represent the aforementioned planetary building material that remained unsampled among meteorites. The necessary enrichment of CC material in *s*-process matter may perhaps be possible as a result of filtering (*26*, *27*) or thermal processing (*28*) of CC dust during its transport into the inner solar system.

However, several lines of evidence indicate that the unsampled planetary building material is not CC dust. First, the isotopic composition of the putative *s*-process–enriched CC dust may be quantified by extrapolation of the NC line to the intersection with the typical Ca, Ti, Cr, and Ni isotopic composition of CC meteorites. This is possible because the isotopes of Ca, Ti, Cr, and Ni are not produced in significant amount in the *s*-process, such that processing of *s*-process carriers would not significantly change the isotopic composition of these elements. However, the projected isotope compositions determined in this manner plot on the NC line in Mo isotope space and not, as would be required, at the intersection of the NC and CC lines (fig. S3). Second, if the isotopic trend among NC meteorites were to reflect the progressive addition of CC dust, then the isotopic compositions of NC bodies would have evolved over time toward more CC-like compositions. However, there is no obvious correlation between isotope anomalies and accretion ages of NC meteorite parent bodies, indicating that the isotope variability among NC meteorites does not reflect a temporal evolution of inner disk composition (*20*). Third, the isotope variability among NC meteorites seems to follow a heliocentric gradient. Although their original formation location is not well known, it is often assumed that enstatite chondrites, which have the lowest ε^94^Mo and ε^96^Zr and highest ε^50^Ti and ε^54^Cr among NC meteorites, formed closer to the Sun than other NC meteorites such as ordinary chondrites or ureilites (*29*). This gradient is also consistent with the higher ε^94^Mo and ε^96^Zr and lower ε^50^Ti and ε^54^Cr of Mars compared to Earth and would also be consistent with the lower ε^50^Ti and ε^54^Cr of HED meteorites, provided that the HED parent body, asteroid Vesta, formed close to its present-day position in the asteroid belt (Fig. 2). Together, these observations suggest that the unsampled *s*-process–enriched material derives from the innermost disk sunward of the formation location of known NC meteorites. This material likely remained unsampled among meteorites because of the low probability for scattering objects from the innermost disk into the asteroid belt, where the parent bodies of meteorites remained stored over the age of the solar system.

### Origin of isotopic difference between Earth and Mars

The average ε^94^Mo of the NC material contributing to Mo in BSE and BSM is more negative for Earth than for Mars, indicating that Earth accreted more of the unsampled, *s*-process–enriched material from the innermost solar system than Mars did. This is consistent with Mars’ formation at greater heliocentric distance than Earth and with our model that material closer to the Sun plots on the multielement NC correlation lines beyond the terrestrial composition. The isotopic difference between Earth and Mars, therefore, likely reflects their formation at different radial locations in the disk. This resulted in different ratios of objects with more positive (such as NC meteorites) and more negative ε^94^Mo (the unsampled material from above) in the NC accretionary mixtures of Earth and Mars, which in turn are reflected in different positions along the NC isotope correlation.

Formation of Earth and Mars from heterogeneous mixtures of NC bodies seems at odds with the observation that for all elements investigated so far, Earth is isotopically very similar to enstatite chondrites (*13*) and that Mars is typically intermediate between enstatite and ordinary chondrites (*15*, *16*). This has been interpreted to reflect accretion of Earth and Mars from isotopically homogeneous materials (*13*, *15*). However, the isotopic link between Earth and enstatite chondrites arises naturally from the multielement isotope correlations among NC meteorites. Once the heterogeneous mixture that made Earth is, by chance, isotopically similar to a given meteorite group for one element, the same isotopic similarity will necessarily hold for all other elements. The same applies to Mars, but at a different position on the NC isotope correlation.

### Mode of terrestrial planet formation in the solar system

A key result of our study is that once a representative set of elements is considered, the isotopic composition of Earth and Mars indicates that both planets accreted only a small fraction of CC material, with a peak at 4% by mass (Fig. 5C). Thus, contrary to some recent studies (*7*, *9*, *10*), we conclude that the terrestrial planets could not have formed by accretion of large masses (~40%) of CC pebbles from the outer solar system. Instead, the terrestrial planets could only have formed by pebble accretion if the vast majority of the pebbles had an NC isotopic composition. To this end, it has been suggested that CC material initially resided far from the Sun and that it reached the terrestrial planet region by radial drift only after some time *t*, before which the planets grew by accreting NC pebbles (*7*). Within this framework, the fraction of CC material in the terrestrial planets would be higher the earlier the change in pebble composition from NC to CC in the terrestrial planet region would have occurred. For instance, to match the purported high CC fraction in the terrestrial planets (*9*, *10*) in this particular model would require this change to have occurred at ~3.8 million years (Ma) after solar system formation (*7*). However, the low CC fraction in the terrestrial planets determined in the present study indicates that this assumed change in pebble composition would have to have occurred at ~5 Ma, which is the assumed lifetime of the disk in this model (*7*). Moreover, for a typical pebble Stokes number of 0.001, the dust reaching the terrestrial planet region at 5 Ma would have originally been located at ~100 astronomical units (AU). However, given that the parent bodies of some CC iron meteorites formed within 1 Ma after the start of the solar system (*30*) and considering that these bodies have been implanted into the asteroid belt during the growth of the giant planets (*8*, *31*), it is implausible that the original boundary between the NC and CC reservoirs was located as far out as ~100 AU. Consequently, the most reasonable and straightforward interpretation is that the low fraction of CC material in the terrestrial planets implies a markedly reduced influx of dust into the inner disk. It has been suggested that a pressure maximum in the disk or the snow line acted as barriers against grain drift and, hence, were responsible for the initial separation of the NC and CC reservoirs (*32*–*34*). However, it is unclear as to whether these transient disk structures were capable of maintaining an efficient separation of the NC and CC reservoirs over the entire lifetime of the disk. Hence, we propose that the most plausible cause for the long-lasting separation of the NC and CC reservoirs is the formation of Jupiter, which has been shown to act as an efficient and persistent barrier against the inward drift of dust and pebbles (*26*, *27*, *35*).

Despite the low bulk CC fraction, Earth and Mars must have accreted some CC bodies during later growth stages, because otherwise, the BSE and BSM would not plot between the NC and CC lines (Fig. 3). The CC contributions to the BSE’s and BSM’s Mo are remarkably similar (Fig. 5B), indicating a common process of CC accretion for both bodies. For instance, although the mixed NC-CC Mo isotopic composition of the BSE may reflect delivery of CC material by the Moon-forming impactor (*18*), a similar process did not occur for Mars. Moreover, our results indicate that CC planetesimals were already present in the inner disk when Mars reached its final mass at ~5 Ma after solar system formation (*36*). We, therefore, suggest that Earth and Mars, as well as other planetary embryos in the inner solar system, most likely accreted CC material through planetesimals, which contributed little overall mass but nevertheless left a significant imprint on their mantle’s Mo isotopic compositions. This is consistent with chemical models for core-mantle differentiation on Earth, which also argue for the accretion of CC planetesimals toward the end of accretion (*37*). Last, we note that the estimate of ~4% CC material in Earth and Mars is sufficient to account for the volatile element inventory of both planets, even under the unlikely condition that all NC material accreted by them was volatile free (*38*–*40*).

The subordinate role of outer solar system material among the accretionary mix of Earth and Mars is consistent with the classic model of terrestrial planet formation by oligarchic growth through collisions among inner solar system planetesimals and planetary embryos, with only little contamination from outer solar system objects. Of note, our estimate of only ~4% CC material in Earth and Mars is consistent with the results of dynamic models in which CC asteroids are scattered into the inner solar system during the growth (*8*) and/or migration (*41*) of Jupiter. This combined with the efficient isolation of the inner disk from inward-drifting dust from the outer solar system required by the isotopic data bears testimony to the crucial role of Jupiter for determining the fundamental process that built terrestrial planets in the solar system.

## MATERIALS AND METHODS

### Samples, sample digestion, and initial chemistry

The 17 martian meteorites of this study were derived from distinct sources of the martian mantle, which were established during the early differentiation of a martian magma ocean. Samples include five depleted shergottites (DaG 476, LAR 12011, NWA 7635, SaU 005, and Tissint), three intermediate shergottites (ALH 77005, EETA 79001, and NWA 7042), four enriched shergottites (LAR 12011, NWA 4864, RBT 04262, and Zagami), three nakhlites (Nakhla, NWA 10153, and MIL 03346), orthopyroxenite ALH 84001, and augite basalt NWA 8159. Individual sample masses ranged from 0.2 to 3.6 g, which combined amount to a total of ~22 g of martian material. Following cleaning of fresh interior pieces by ultrasonication in ethanol, samples were crushed in an agate mortar, and sample powders were digested in Savillex perfluoralkoxy (PFA) beakers with a 2:1 mixture of HF:HNO_3_ on a hotplate at 130° to 150°C for 2 to 5 days. Titanium, Zr, and Mo (this study), as well as Cr (*42*), and Nd and W (*43*), were then separated from the sample matrix using ion exchange chromatography following our previously established procedures (*18*, *24*, *44*–*47*). Samples were loaded onto columns filled with AG1X8 (4 ml, 200 to 400 mesh) in 0.5M HCl–0.5M HF (25 ml). Samples larger than 0.5 g were split over two or more columns. Following elution of matrix elements, Cr and rare earth elements (REE) with further addition of 0.5 M HCl–0.5 M HF (10 ml), Ti, Zr, and Hf were eluted with 10 ml of 8 M HCl–0.05 M HF. Last, W and Mo were rinsed off the columns in 15 ml of 6 M HCl–1 M HF and 10 ml of 3 M HNO_3_, respectively. Two large samples (3.56 g of DaG 476 and 3.42 g of Tissint) were exclusively processed for this study and were digested for 7 days in HF-HNO_3_, followed by HCl-HNO_3_. In addition, for these two samples, each 0.5-g split was loaded in 75 ml of 0.5 M HCl–0.5 M HF on the column, and the Ti, Zr, Hf elution step was omitted, such that these elements were eluted together with W. For all samples split over two or more columns, the individual matrix (+Cr, REE), Ti-Zr-Hf, Mo, and W cuts from each column were combined after the first column.

### Titanium cleanup chemistry and isotope measurements

The Ti-Zr-Hf cuts of eight shergottites (DaG 476, LAR 12011, NWA 7635, SaU 005, Tissint, ALH 77005, EETA 79001, and NWA 7042), orthopyroxenite ALH84001, and augite basalt NWA 8159 were dried and redissolved in 12 M HNO_3_. They were subsequently passed through a two-stage ion exchange procedure involving TODGA (*N*,*N*,*N*′,*N*′-tetraoctyl diglycolamide) (2 ml) and AG1X8 (0.8 ml, 200 to 400 mesh) ion exchange resins (*48*) to separate Ti from Zr and Hf. Titanium isotope measurements were made on a Thermo Fisher Scientific Neptune Plus multicollector inductively coupled plasma mass spectrometer (MC-ICPMS) in high-resolution mode at the Institut für Planetologie in Münster (*45*). Solutions containing about 400 ng/g Ti were introduced through a Cetac Aridus II desolvating system, resulting in a total ion beam of ~4.5 × 10^−10^ A. Each Ti isotope measurement consisted of two lines of data acquisition. After 30-s baseline integrations (deflected beam), ion beams on all Ti isotopes as well as ^51^V and ^53^Cr were measured in line 1 in blocks of 40 cycles of 4.2 s each, whereas in line 2, ion beams on ^44^Ca and all the Ti isotopes were measured in 20 cycles of 4.2-s integrations each. Instrumental mass bias was corrected using the exponential law and ^49^Ti/^47^Ti = 0.749766. Titanium isotope compositions are reported as ε^i^Ti values relative to the mean of bracketing measurements of the OL-Ti (Origins Lab) standard, where ε^i^Ti = [(^i^Ti/^47^Ti)_sample_/(^i^Ti/^47^Ti)_standard_ − 1] × 10^4^. Results were normalized to the mean of the terrestrial standards (BHVO-2, BIR1a, and OL-Ti) processed through the chemistry along with the samples. The latter was done because we observed a slight offset in ε^46^Ti (−0.08 ± 0.05, 95% confidence interval, *N* = 33) and ε^50^Ti (−0.05 ± 0.04) between the processed terrestrial rock samples and standards and the unprocessed OL-Ti bracketing standard. These offsets might be caused by an improper mass fractionation correction assuming the exponential law or unaccounted matrix effects and have been observed (and corrected this way) in other high-precision Ti isotope studies before (*49*). The uncertainty introduced by this correction was propagated into the analytical uncertainties of the Ti isotope measurements for all samples by quadratic addition. The external reproducibility (2 SDs) of the standard measurements during the course of this study was ±0.26 ε^46^Ti, ±0.20 ε^48^Ti, and ± 0.31 ε^50^Ti. The Ti isotope compositions of the martian meteorites analyzed in this study are provided in table S1. All 10 samples of this study have indistinguishable Ti isotopic compositions with a mean ε^50^Ti of −0.42 ± 0.05, which provides the current best estimate for the Ti isotopic composition of Mars.

### Zirconium cleanup chemistry and isotope measurement

For samples with sufficiently high Zr contents, the Zr-Hf cuts from the first column of the Ti chemistry were evaporated to dryness and redissolved in 2 ml of 3 M HNO_3_ and 1 weight % (wt %) H_2_O_2_ for further purification following the procedure of (*50*). Sample solutions were loaded onto PFA shrink columns filled with Eichrom LN spec resin (100 to 150 mesh) for separation of Zr from residual Fe, Ti, and Hf. First, Ti was eluted with 10 ml of 3 M HNO_3_ and 1 wt % H_2_O_2_ in 2-ml incremental steps, which was followed by 2 ml of 0.28 M HNO_3_ to wash off residual H_2_O_2_ from the resin. After rinsing with additional 10 ml of 0.5 M HNO_3_–0.06 M HF, Zr was collected in 24 ml of 0.5 M HNO_3_–0.06 M HF, before Hf was eluted with 6 ml of 0.56 M HNO_3_–0.3 M HF. The final Zr cuts were treated with concentrated HNO_3_ and redissolved in 0.5 M HNO_3_–0.01 M HF for isotope measurements. The purified Zr cuts had Mo/Zr < 1 × 10^−4^, Ru/Zr < 1 × 10^−4^, Ti/Zr < 0.1, Fe/Zr < 0.01, and Hf/Zr < 0.01, all of which are well below the thresholds required for accurate of interference corrections. Yields of the entire separation procedure were >80%. Total procedural blanks were <1 ng of Zr and were insignificant, given that more than 10 μg of Zr was processed for each sample. Zirconium isotope measurements were performed using the Neptune Plus MC-ICPMS at the Institut für Planetologie, using Jet sampler and H skimmer cones. Samples were introduced as solutions (~200 ng/g) using a Cetac Aridus II desolvator and a Savillex C-flow nebulizer with an uptake rate of 50 μl/min, resulting in a total ion beam intensity of 6 × 10^−10^ to 8 × 10^−10^ A. Oxide rates were adjusted to <1.5% CeO/Ceni, and samples and standards were matched in concentration to within <15%. Each measurement consisted of 30-s baseline integrations (deflected beam) followed by 200 cycles Zr isotope ratio measurements of 4.2 s each. Ion beams on all Zr masses (90, 91, 92, 94, and 96) were measured using Faraday cups connected to 10^11^-ohm feedback resistors, and ion beams on masses 95 and 99 were measured using Faraday cups connected to 10^12^-ohm feedback resistors to monitor potential isobaric interferences of Mo and Ru on various Zr isotopes. Instrumental mass bias was corrected by internal normalization to ^94^Zr/^90^Zr = 0.3381 and using the exponential law. The Zr isotope data are reported as ε^i^Zr values relative to the mean composition measured for the NIST SRM 3169 Zr standard that was analyzed bracketing the sample measurements, where ε^i^Zr = [(^i^Zr/^90^Zr)_sample_/(^i^Zr/^90^Zr)_standard_ − 1] × 10^4^. The Zr isotopic composition of two terrestrial rock standards (BHVO-2 and BCR-2) processed along with the samples are indistinguishable from the solution standard. The Zr isotope data of the martian meteorites and the terrestrial basalt standards are provided in the table S2. All martian meteorites have indistinguishable Zr isotope anomalies with a mean ε^96^Zr = 0.28 ± 0.03. This is the first report of Zr isotope data for martian meteorites and as such is the current best estimate for the Zr isotopic composition of Mars.

### Molybdenum cleanup chemistry and isotope measurement

Except for Tissint, the Mo cuts of the 17 individual sample digestions common to this study (*43*) did not contain enough Mo (~100 ng) for individual high-precision Mo isotope measurements. The Mo cuts of these samples were, therefore, combined to obtain a total of four samples (Tissint-1, Mars-A, Mars-B, and Mars-C). Combining the Mo cuts from different martian samples is possible because no nucleosynthetic isotope heterogeneity is expected among different martian meteorites. This is because any potential heterogeneity resulting from accretion of isotopically heterogeneous building blocks should have been erased by subsequent melting of the martian mantle and homogenization within a martian magma ocean. This is consistent with the lack of resolved Zr and Ti isotope variations among individual martian meteorites derived from distinct mantle sources observed in this study (see above). Mars-A consists of the combined Mo cuts of the depleted shergottites SaU 005 and DaG 476; Mars-B consists of the combined Mo cuts of the intermediate shergottites ALH 77005, EETA 79001, and NWA 7042; and Mars-C consists of the combined Mo cuts of the remaining 11 samples of the Nd-W isotope study of (*43*) (NWA 8159, RBT 04262, NWA 4864, LAR 12011, LAR 12095, ALH 84001, NWA 7635, Zagami, Nakhla, NWA 10153, and MIL 03346). Together with the larger DaG 476 and Tissint samples exclusively digested for this study, this resulted in a total of six martian Mo samples (Tissint-1, Mars-A, Mars-B, Mars-C, DaG 476, and Tissint-2) on which a Mo cleanup chemistry was performed. The purification of Mo followed our established two-stage ion exchange procedure using AG1X8 (2 ml, 100 to 200 mesh) and TRU (1 ml) ion exchange resin (*18*, *30*, *51*). After the ion exchange chemistry, the purified Mo cuts were treated with inverse aqua regia to destroy organic compounds before they were taken up in 0.5 M HNO_3_–0.01 M HF for isotope measurement. The Mo yields for the separation were between 65 and 82%, and the procedural blanks ranged from 3 to 16 ng. This corresponds to 3 to 9% of the Mo recovered from each of sample and, given the overall small Mo isotope anomalies in the samples, had no significant effect on measured Mo isotope compositions. The terrestrial rock standards JA-2 and W-2a, which were processed through the analytical procedure along with the martian samples, yielded the terrestrial Mo isotopic composition [reported in (*18*)], testifying to the accuracy of the analytical methods. The Mo isotopic compositions were measured using a Thermo Fisher Scientific Neptune Plus MC-ICP-MS at the Institut für Planetologie. Samples were introduced as Mo solutions (100 ng/g) using a Savillex C-Flow PFA nebulizer (uptake rate ~50 μl/min) connected to a Cetac Aridus II desolvator, resulting in total ion beam intensities of ~1.1 × 10^−10^ A. Each measurement consisted of 40 on-peak baseline integrations, followed by 100 cycles of isotope ratio measurements of 8.4 s each. Instrumental mass bias was corrected by internal normalization to ^98^Mo/^96^Mo = 1.453173 and using the exponential law. Isobaric interferences of Zr and Ru on Mo masses were corrected by monitoring ^91^Zr and ^99^Ru. The maximum corrections were < 0.5 ε and were all well within the range of reliable and accurate interference corrections (*51*). The Mo isotope data are reported as ε^i^Mo values relative to the mean of bracketing Alfa Aesar Mo standard measurements, where ε^i^Mo = [(^i^Mo/^96^Mo)_sample_/(^i^Mo/^96^Mo)_standard_ − 1] × 10^4^. The external reproducibility (2 SDs) of the standard measurement during the course of this study was ±0.29 ε^92^Mo, ±0.25 ε^94^Mo, ±0.18 ε^95^Mo, ±0.15 ε^97^Mo, and ± 0.14 ε^100^Mo. The Mo isotope data of the martian samples are reported in table S3. Within the external reproducibility of the measurements, all martian meteorites have identical Mo isotope compositions and reveal resolved Mo isotope anomalies relative to the standard and terrestrial samples. The mean Mo isotopic composition of the martian meteorites is consistent with an *s*-process deficit relative to Earth’s mantle and, given that the samples of this study derive from different portions of the martian mantle, provides the current best estimate for the Mo isotopic composition of BSM. Although there is some scatter in the individual measurements, in a diagram of ε^95^Mo versus ε^94^Mo, the average BSM composition plots between the NC and CC lines (Fig. 3). Together, these data indicate that the BSM’s Mo derives from both the NC and CC reservoirs, where the fraction of CC-derived Mo, as calculated from its position between the NC and CC lines using the lever rule, is 0.4 ± 0.3 (2σ). As a siderophile element, the BSM’s Mo predominantly records the last ~20% of accretion, because the Mo from earlier accretion stages has been removed to the core. Thus, the Mo isotopic data indicate that Mars accreted some CC material during the last ~20% of its growth, but the fraction of CC material accreted by Mars during its entire accretion history cannot be determined in this manner. More precise and robust constraints on how much CC material was accreted by Mars can be obtained by considering its isotopic composition in multielement space (see below and main text).

### Isotopic mixing model for Earth and Mars

As discussed in the main text, the building blocks of Earth (or Mars) can be reduced to three main components: average NC material contributing to the BSE’s Mo budget (denoted NC_Late_), average NC material not contributing to the BSE’s Mo (denoted NC_Early_), and CC material. The indexes “Early” and “Late” reflect the siderophile nature of Mo, which means that the material contributing to the BSE’s Mo likely accreted later than the material that did not contribute to the final Mo budget of the BSE (that is, the Mo from these objects was entirely removed to Earth’s core). These three components come with five unknowns: the ε^94^Mo values of NC_Late_ and NC_Early_, denoted ε^94^Mo^NCLate^ and ε^94^Mo^NCEarly^, the fraction $f_{\text{Mo}}^{\text{NCLate}}$ of the BSE’s Mo delivered by NC_Late_, and the fractions of CC and NC_Late_ in the bulk Earth, $f_{\text{BULK}}^{\text{CC}}$ and $f_{\text{BULK}}^{\text{NCLate}}$. Note that $f_{\text{Mo}}^{\text{NCLate}}$ should not be confused with the fraction of Earth’s mass that accreted “late,” but merely refers to the fraction of a mantle’s NC-derived Mo that has been delivered late. We also note that because for all isotope anomalies, NC meteorites plot on the NC line, once ε^94^Mo^NCLate^ and ε^94^Mo^NCEarly^ are known, their isotope anomalies for all other elements are known as well.

To determine ε^94^Mo^NCLate^ and $f_{\text{Mo}}^{\text{NCLate}}$, we use two equations for ε^94^Mo and ε^95^Mo. These are

$$\varepsilon^{94}{\text{Mo}}^{\text{NCLate}}f_{\text{Mo}}^{\text{NCLate}}+(1-f_{\text{Mo}}^{\text{NCLate}})\varepsilon^{94}{\text{Mo}}^{\text{CC}}=\varepsilon^{94}{\text{Mo}}^{\text{BSE}}$$ (1)

$$(a_{95\text{Mo}}\varepsilon^{94}{\text{Mo}}^{\text{NCLate}}+b_{95\text{Mo}})f_{\text{Mo}}^{\text{NCLate}}+(1-f_{\text{Mo}}^{\text{NCLate}})\varepsilon^{95}{\text{Mo}}^{\text{CC}}=\varepsilon^{95}{\text{Mo}}^{\text{BSE}}$$ (2)

where ε^94^Mo^CC^, ε^95^Mo^CC^, ε^94^Mo^BSE^, and ε^95^Mo^BSE^ are the average values of ε^94^Mo and ε^95^Mo for CC meteorites and BSE, respectively, and *a*_95Mo_ and *b*_95Mo_ are the coefficients of the NC line in the ε^95^Mo-ε^94^Mo plane. This system of equation gives the solution

$$\begin{array}{c} \varepsilon^{94}{\text{Mo}}^{\text{NCLate}}=\\ (b_{95\text{Mo}}\varepsilon^{94}{\text{Mo}}^{\text{CC}}-b_{95\text{Mo}}\varepsilon^{94}{\text{Mo}}^{\text{BSE}}-\varepsilon^{94}{\text{Mo}}^{\text{CC}}\varepsilon^{95}{\text{Mo}}^{\text{BSE}}+\varepsilon^{95}{\text{Mo}}^{\text{CC}}\varepsilon^{94}{\text{Mo}}^{\text{BSE}})/\\ \left(-a_{95\text{Mo}}\varepsilon^{94}{\text{Mo}}^{\text{CC}}+a_{95\text{Mo}}\varepsilon^{94}{\text{Mo}}^{\text{BSE}}-\varepsilon^{95}{\text{Mo}}^{\text{BSE}}+\varepsilon^{95}{\text{Mo}}^{\text{CC}}\right) \end{array}$$ (3)

$$\begin{array}{c} f_{\text{Mo}}^{\text{NCLate}}=(-a_{95\text{Mo}}\varepsilon^{94}{\text{Mo}}^{\text{CC}}+a_{95\text{Mo}}\varepsilon^{94}{\text{Mo}}^{\text{BSE}}-\varepsilon^{95}{\text{Mo}}^{\text{BSE}}+\varepsilon^{95}{\text{Mo}}^{\text{CC}})/\\ \left(-a_{95\text{Mo}}\varepsilon^{94}{\text{Mo}}^{\text{CC}}-b_{95\text{Mo}}+\varepsilon^{95}{\text{Mo}}^{\text{CC}}\right) \end{array}$$ (4)

To determine ε^94^Mo^NCEarly^, $f_{\text{BULK}}^{\text{CC}}$, and $f_{\text{BULK}}^{\text{NCLate}}$, let us start by considering two equations for the isotope anomalies of two lithophile elements, such as ε^54^Cr and ε^96^Zr

$$\begin{array}{c} (a_{54\text{Cr}}\varepsilon^{94}{\text{Mo}}^{\text{NCLate}}+b_{54\text{Cr}})f_{\text{BULK}}^{\text{NCLate}}+f_{\text{BULK}}^{\text{CC}}\varepsilon^{54}{\text{Cr}}^{\text{CC}}+\\ (1-f_{\text{BULK}}^{\text{NCLate}}-f_{\text{BULK}}^{\text{CC}})(a_{54\text{Cr}}\varepsilon^{94}{\text{Mo}}^{\text{NCEarly}}+b_{54\text{Cr}})=\varepsilon^{54}{\text{Cr}}^{\text{BSE}} \end{array}$$ (5)

$$\begin{array}{c} (a_{96\text{Zr}}\varepsilon^{94}{\text{Mo}}^{\text{NCLate}}+b_{96\text{Zr}})f_{\text{BULK}}^{\text{NCLate}}+f_{\text{BULK}}^{\text{CC}}\varepsilon^{96}{\text{Zr}}^{\text{CC}}+\\ (1-f_{\text{BULK}}^{\text{NCLate}}-f_{\text{BULK}}^{\text{CC}})(a_{96\text{Zr}}\varepsilon^{94}{\text{Mo}}^{\text{NCEarly}}+b_{96\text{Zr}})=\varepsilon^{96}{\text{Zr}}^{\text{BSE}} \end{array}$$ (6)

where the meaning of the variables is like before, just substituting ^95^Mo with ^54^Cr or ^96^Zr. These two equations have the same functional form, even if their coefficients are different. Thus, instead of giving two unknowns as function of the third, there is one value of $f_{\text{BULK}}^{\text{CC}}$ that makes the two equations linearly dependent (that is, equivalent to each other). This is

$$\begin{array}{c} f_{\text{Bulk}}^{\text{CC}}=(a_{54\text{Cr}}b_{96\text{Zr}}-a_{54\text{Cr}}\varepsilon^{96}{\text{Zr}}^{\text{BSE}}-a_{96\text{Zr}}b_{54\text{Cr}}+a_{96\text{Zr}}\varepsilon^{54}{\text{Cr}}^{\text{BSE}})/\\ \left(a_{54\text{Cr}}b_{96\text{Zr}}-a_{54\text{Cr}}\varepsilon^{96}{\text{Zr}}^{\text{CC}}-a_{96\text{Zr}}b_{54\text{Cr}}+a_{96\text{Zr}}\varepsilon^{54}{\text{Cr}}^{\text{CC}}\right) \end{array}$$ (7)

In this equation, $f_{\text{Bulk}}^{\text{CC}}$ represents the mass fraction of a lithophile element that was added to Earth by CC material. It can be safely assumed that this is the fraction of Earth’s mass delivered by CC bodies. This assumption would not have been valid only if most of the NC and CC bodies accreted by Earth (and Mars) were not chondritic in terms of the mass fraction of lithophile elements (i.e., because they were accreted as discrete cores or mantle debris from differentiated planetesimals). However, given that the Fe/Si ratio of the Earth is very close to chondritic, this can be excluded. For the value of $f_{\text{BULK}}^{\text{CC}}$ given in Eq. 7, each of Eqs. 5 and 6 gives the same relationship ε^94^Mo^NCEarly^ ($f_{\text{BULK}}^{\text{NCLate}}$). Thus, it is not possible to determine ε^94^Mo^NCEarly^ and $f_{\text{BULK}}^{\text{NCLate}}$ separately. Instead, for any other value of $f_{\text{BULK}}^{\text{CC}}$, Eqs. 5 and 6 do not have any common intersection. Adding a third equation for another lithophile element (e.g., ε^145^Nd, name it Eq. 8) would not improve the situation because it would have the same functional form as Eqs. 5 and 6. Ideally, the same value of $f_{\text{BULK}}^{\text{CC}}$ would render the three equations equivalent to each other. In practice, given the errors in the various coefficients, the value of $f_{\text{BULK}}^{\text{CC}}$ obtained by considering Eqs. 5 and 6, 6 and 8, or 5 and 8 will be close, but not identical. Thus, we will only consider Eqs. 5 and 6 and use Eqs. 6 and 8 for control.

Because all coefficients in Eqs. 1, 2, 5, and 6 have uncertainties, it does not make sense to use these equations to derive a single value for ε^94^Mo^NCLate^, $f_{\text{MO}}^{\text{NCLate}}$, and $f_{\text{BULK}}^{\text{CC}}$. Thus, we used a Monte Carlo approach instead. In each trial, we pick a value for each coefficient within its Gaussian uncertainty, up to 3σ from its mean value. Note that correlated uncertainties were taken into account. The uncertainties of the *a* and *b* coefficients of each NC line (say *a*_54Cr_ and *b*_54Cr_) are correlated because the line has to pass through the data points of NC meteorites. We impose that the NC line in the ε^95^Mo-ε^94^Mo plot passes through the point (ε^94^Mo = 0.5 and ε^95^Mo = 0.206), that in the ε^54^Cr-ε^94^Mo plot passes through (ε^94^Mo = 0.75 and ε^54^Cr = −0.483), that in the ε^96^Zr-ε^94^Mo plot passes through (ε^94^Mo = 0.7 and ε^96^Zr = 0.4), and that in the ε^145^Nd-ε^94^Mo plot passes through (ε^94^Mo = 0.54 and ε^145^Nd = 0.05). These points are chosen at the locations where the data constraining the NC lines are the tightest. Another correlation exists between the value of ε^94^Mo^CC^ and ε^95^Mo^CC^, which is given by the CC line (*18*, *51*). The mean values and the 1σ uncertainties of all independent quantities considered in the Monte Carlo calculation are provided in table S4. Those concerning the isotopic anomalies of CC meteorites are computed from the mean and root mean square of the mean values of all types of CC meteorites (data S1). In the Monte Carlo calculations, we performed 5 million trials. The results for ε^94^Mo^NCLate^, $f_{\text{Mo}}^{\text{NCLate}}$, and $f_{\text{BULK}}^{\text{CC}}$ are then binned in the intervals [−3:1], [0:1], and [0:1], and the resulting PDFs are reported in Fig. 5. As a control, we repeated the Monte Carlo calculation using the equation for ε^145^Nd instead of ε^96^Zr. The resulting PDF for $f_{\text{BULK}}^{\text{CC}}$ is very similar. Instead of having a maximum at $f_{\text{BULK}}^{\text{CC}}$ = 0.04 it monotonically decays from 0. Only 7.7% of the trials give $f_{\text{BULK}}^{\text{CC}}$ > 0.2 (instead of 2.5% in the case of Fig. 5).

We have also applied the same method for Mars. The values of the isotopic anomalies for BSM are reported in table S5. The PDFs for the unknowns ε^94^Mo^NCLate^, $f_{\text{Mo}}^{\text{NCLate}}$, and $f_{\text{BULK}}^{\text{CC}}$ are shown in Fig. 5. Our results show that the value of $f_{\text{Mo}}^{\text{NCLate}}$ for Mars is very similar to that for the Earth, indicating that the CC contributions to Mo in the mantles of both planets are comparable. Instead, the CC fraction to bulk Mars is much more restricted than that of the Earth, not exceeding 0.1. The value of ε^94^Mo^NCLate^ is larger for Mars than for the Earth, as can also be deduced graphically from Fig. 5, meaning—in our interpretation—that Mars did not reach material as close to the Sun as the Earth did. This makes sense given that Mars is 50% further away.

## Supplementary Materials

### This PDF file includes:

Figs S1 to S3 Tables S1 to S5 Legend for data S1 References

## References

[1] J. E. Chambers, G. W. Wetherill, Making the terrestrial planets: N-body integrations of planetary embryos in three dimensions. Icarus 136, 304–327 (1998).

[2] M. Lambrechts, A. Johansen, Rapid growth of gas-giant cores by pebble accretion. Astron. Astrophys. 544, A32 (2012).

[3] C. W. Ormel, H. H. Klahr, The effect of gas drag on the growth of protoplanets. Analytical expressions for the accretion of small bodies in laminar disks. Astron. Astrophys. 520, A43 (2010).

[4] M. Lambrechts, A. Johansen, Forming the cores of giant planets from the radial pebble flux in protoplanetary discs. Astron. Astrophys. 572, A107 (2014).

[5] H. F. Levison, K. A. Kretke, M. J. Duncan, Growing the gas-giant planets by the gradual accumulation of pebbles. Nature 524, 322–324 (2015). 26289203 10.1038/nature14675

[6] H. F. Levison, K. A. Kretke, K. J. Walsh, W. F. Bottke, Growing the terrestrial planets from the gradual accumulation of submeter-sized objects. Proc. Natl. Acad. Sci. 112, 14180–14185 (2015). 26512109 10.1073/pnas.1513364112 PMC4655528

[7] A. Johansen, T. Ronnet, M. Bizzarro, M. Schiller, M. Lambrechts, Å. Nordlund, H. Lammer, A pebble accretion model for the formation of the terrestrial planets in the Solar System. Sci. Adv. 7, eabc0444 (2021). 33597233 10.1126/sciadv.abc0444 PMC7888959

[8] S. N. Raymond, A. Izidoro, Origin of water in the inner Solar System: Planetesimals scattered inward during Jupiter and Saturn’s rapid gas accretion. Icarus 297, 134–148 (2017).

[9] M. Schiller, M. Bizzarro, J. Siebert, Iron isotope evidence for very rapid accretion and differentiation of the proto-Earth. Sci. Adv. 6, eaay7604 (2020). 32095530 10.1126/sciadv.aay7604 PMC7015677

[10] M. Schiller, M. Bizzarro, V. A. Fernandes, Isotopic evolution of the protoplanetary disk and the building blocks of Earth and the Moon. Nature 555, 507–510 (2018). 29565359 10.1038/nature25990 PMC5884421

[11] P. H. Warren, Stable-isotopic anomalies and the accretionary assemblage of the Earth and Mars: A subordinate role for carbonaceous chondrites. Earth Planet. Sci. Lett. 311, 93–100 (2011).

[12] C. Burkhardt, T. Kleine, F. Oberli, A. Pack, B. Bourdon, R. Wieler, Molybdenum isotope anomalies in meteorites: Constraints on solar nebula evolution and origin of the Earth. Earth Planet. Sci. Lett. 312, 390–400 (2011).

[13] N. Dauphas, The isotopic nature of the Earth’s accreting material through time. Nature 541, 521–524 (2017). 28128239 10.1038/nature20830

[14] K. R. Bermingham, E. A. Worsham, R. J. Walker, New insights into Mo and Ru isotope variation in the nebula and terrestrial planet accretionary genetics. Earth Planet. Sci. Lett. 487, 221–229 (2018). 30880823 10.1016/j.epsl.2018.01.017 PMC6417891

[15] R. Brasser, N. Dauphas, S. J. Mojzsis, Jupiter’s influence on the building blocks of Mars and Earth. Geophys. Res. Lett. 45, 5908–5917 (2018).

[16] H. Tang, N. Dauphas, ^60^Fe–^60^Ni chronology of core formation in Mars. Earth Planet. Sci. Lett. 390, 264–274 (2014).

[17] K. Mezger, M. Schönbächler, A. Bouvier, Accretion of the Earth—Missing components? Space Sci. Rev. 216, 27 (2020).

[18] G. Budde, C. Burkhardt, T. Kleine, Molybdenum isotopic evidence for the late accretion of outer solar system material to Earth. Nat. Astron. 3, 736–741 (2019).

[19] M. Fischer-Gödde, T. Kleine, Ruthenium isotopic evidence for an inner solar system origin of the late veneer. Nature 541, 525–527 (2017). 28128236 10.1038/nature21045

[20] F. Spitzer, C. Burkhardt, G. Budde, T. S. Kruijer, A. Morbidelli, T. Kleine, Isotopic evolution of the inner solar system inferred from molybdenum isotopes in meteorites. Astrophys. J. 898, L2 (2020).

[21] A. Trinquier, T. Elliott, D. Ulfbeck, C. Coath, A. N. Krot, M. Bizzarro, Origin of nucleosynthetic isotope heterogeneity in the solar protoplanetary disk. Science 324, 374–376 (2009). 19372428 10.1126/science.1168221

[22] C. Burkhardt, N. Dauphas, H. Tang, M. Fischer-Gödde, L. Qin, J. H. Chen, S. S. Rout, A. Pack, P. R. Heck, D. A. Papanastassiou, In search of the Earth-forming reservoir: Mineralogical, chemical, and isotopic characterizations of the ungrouped achondrite NWA 5363/NWA 5400 and selected chondrites. Meteorit. Planet. Sci. 52, 807–826 (2017).

[23] C. D. Williams, M. E. Sanborn, C. Defouilloy, Q. Z. . Yin, N. T. Kita, D. S. Ebel, A. Yamakawa, K. Yamashita, Chondrules reveal large-scale outward transport of inner solar system materials in the protoplanetary disk. Proc. Natl. Acad. Sci. U.S.A. 117, 23426–23435 (2020). 32900966 10.1073/pnas.2005235117 PMC7519341

[24] C. Burkhardt, L. E. Borg, G. A. Brennecka, Q. R. Shollenberger, N. Dauphas, T. Kleine, A nucleosynthetic origin for the Earth’s anomalous ^142^Nd composition. Nature 537, 394–398 (2016). 27629643 10.1038/nature18956 PMC5026299

[25] T. Kleine, G. Budde, C. Burkhardt, T. S. Kruijer, E. A. Worsham, A. Morbidelli, F. Nimmo, The noncarbonaceous-carbonaceous meteorite dichotomy. Space Sci. Rev. 216, 55 (2020).

[26] T. Haugbølle, P. Weber, D. P. Wielandt, P. Benítez-Llambay, M. Bizzarro, O. Gressel, M. E. Pessah, Probing the protosolar disk using dust filtering at gaps in the early solar system. Astron. J. 158, 55 (2019).

[27] P. Weber, P. Benítez-Llambay, O. Gressel, L. Krapp, M. E. Pessah, Characterizing the variable dust permeability of planet-induced gaps. Astrophys. J. 854, 153 (2018).

[28] M. Ek, A. C. Hunt, M. Lugaro, M. Schönbächler, The origin of *s*-process isotope heterogeneity in the solar protoplanetary disk. Nat. Astron. 4, 273–281 (2020).

[29] S. J. Desch, A. Kalyaan, C. M. O.’. D. Alexander, The effect of Jupiter’s formation on the distribution of refractory elements and inclusions in meteorites. Astrophys. J. Suppl. Ser. 238, 11 (2018).

[30] T. S. Kruijer, C. Burkhardt, G. Budde, T. Kleine, Age of Jupiter inferred from the distinct genetics and formation times of meteorites. Proc. Natl. Acad. Sci. U.S.A. 114, 6712–6716 (2017). 28607079 10.1073/pnas.1704461114 PMC5495263

[31] K. J. Walsh, A. Morbidelli, S. N. Raymond, D. P. O’Brien, A. M. Mandell, A low mass for Mars from Jupiter’s early gas-driven migration. Nature 475, 206–209 (2011). 21642961 10.1038/nature10201

[32] R. Brasser, S. J. Mojzsis, The partitioning of the inner and outer Solar System by a structured protoplanetary disk. Nat. Astron. 4, 492–499 (2020).

[33] S. Charnoz, G. Avice, R. Hyodo, F. C. Pignatale, M. Chaussidon, Forming pressure traps at the snow line to isolate isotopic reservoirs in the absence of a planet. A&A 652, A35 (2021).

[34] T. Lichtenberg, J. Drążkowska, M. Schönbächler, G. J. Golabek, T. O. Hands, Bifurcation of planetary building blocks during Solar System formation. Science 371, 365–370 (2021). 33479146 10.1126/science.abb3091

[35] M. Lambrechts, A. Johansen, A. Morbidelli, Separating gas-giant and ice-giant planets by halting pebble accretion. Astron. Astrophys. 572, A35 (2014).

[36] N. Dauphas, A. Pourmand, Hf-W-Th evidence for rapid growth of Mars and its status as a planetary embryo. Nature 473, 489–492 (2011). 21614076 10.1038/nature10077

[37] D. C. Rubie, S. A. Jacobson, A. Morbidelli, D. P. O’Brien, E. D. Young, J. de Vries, F. Nimmo, H. Palme, D. J. Frost, Accretion and differentiation of the terrestrial planets with implications for the compositions of early-formed Solar System bodies and accretion of water. Icarus 248, 89–108 (2015).

[38] B. Marty, The origins and concentrations of water, carbon, nitrogen and noble gases on Earth. Earth Planet. Sci. Lett. 313-314, 56–66 (2012).

[39] L. Piani, Y. Marrocchi, T. Rigaudier, L. G. Vacher, D. Thomassin, B. Marty, Earth’s water may have been inherited from material similar to enstatite chondrite meteorites. Science 369, 1110–1113 (2020). 32855337 10.1126/science.aba1948

[40] K. Lodders, B. Fegley, An oxygen isotope model for the composition of Mars. Icarus 126, 373–394 (1997).

[41] D. P. O’Brien, K. J. Walsh, A. Morbidelli, S. N. Raymond, A. M. Mandell, Water delivery and giant impacts in the ‘Grand Tack’ scenario. Icarus 239, 74–84 (2014).

[42] T. S. Kruijer, L. E. Borg, J. Wimpenny, C. K. Sio, Onset of magma ocean solidification on Mars inferred from Mn-Cr chronometry. Earth Planet. Sci. Lett. 542, 116315 (2020).

[43] T. S. Kruijer, T. Kleine, L. E. Borg, G. A. Brennecka, A. J. Irving, A. Bischoff, C. B. Agee, The early differentiation of Mars inferred from Hf-W chronometry. Earth Planet. Sci. Lett. 474, 345–354 (2017).

[44] L. E. Borg, G. A. Brennecka, S. J. K. Symes, Accretion timescale and impact history of Mars deduced from the isotopic systematics of martian meteorites. Geochim. Cosmochim. Acta 175, 150–167 (2016).

[45] S. Gerber, C. Burkhardt, G. Budde, K. Metzler, T. Kleine, Mixing and transport of dust in the early solar nebula as inferred from titanium isotope variations among chondrules. Astrophys. J. Lett. 841, L17 (2017).

[46] T. S. Kruijer, T. Kleine, M. Fischer-Gödde, P. Sprung, Lunar tungsten isotopic evidence for the late veneer. Nature 520, 534–537 (2015). 25855296 10.1038/nature14360

[47] J. Render, G. A. Brennecka, Isotopic signatures as tools to reconstruct the primordial architecture of the Solar System. Earth Planet. Sci. Lett. 555, 116705 (2021).

[48] J. J. Zhang, N. Dauphas, A. M. Davis, A. Pourmand, A new method for MC-ICPMS measurement of titanium isotopic composition: Identification of correlated isotope anomalies in meteorites. J. Anal. At. Spectrom. 26, 2197–2205 (2011).

[49] J. J. Zhang, N. Dauphas, A. M. Davis, I. Leya, A. Fedkin, The proto-Earth as a significant source of lunar material. Nat. Geosci. 5, 251–255 (2012).

[50] R. Bast, E. E. Scherer, P. Sprung, K. Mezger, M. Fischer-Gödde, S. Taetz, M. Böhnke, H. Schmid-Beurmann, C. Münker, T. Kleine, G. Srinivasan, Reconciliation of the excess ^176^Hf conundrum in meteorites: Recent disturbances of the Lu-Hf and Sm-Nd isotope systematics. Geochim. Cosmochim. Acta 212, 303–323 (2017).

[51] G. Budde, C. Burkhardt, G. A. Brennecka, M. Fischer-Gödde, T. S. Kruijer, T. Kleine, Molybdenum isotopic evidence for the origin of chondrules and a distinct genetic heritage of carbonaceous and non-carbonaceous meteorites. Earth Planet. Sci. Lett. 454, 293–303 (2016).

[52] T. Hopp, G. Budde, T. Kleine, Heterogeneous accretion of Earth inferred from Mo-Ru isotope systematics. Earth Planet. Sci. Lett. 534, 116065 (2020).

[53] C. Burkhardt, N. Dauphas, U. Hans, B. Bourdon, T. Kleine, Elemental and isotopic variability in solar system materials by mixing and processing of primordial disk reservoirs. Geochim. Cosmochim. Acta 261, 145–170 (2019).

[54] Z. A. Torrano, D. L. Schrader, J. Davidson, R. C. Greenwood, D. R. Dunlap, M. Wadhwa, The relationship between CM and CO chondrites: Insights from combined analyses of titanium, chromium, and oxygen isotopes in CM, CO, and ungrouped chondrites. Geochim. Cosmochim. Acta 301, 70–90 (2021). 34316079 10.1016/j.gca.2021.03.004 PMC8312627

[55] G. A. Brennecka, C. Burkhardt, G. Budde, T. S. Kruijer, F. Nimmo, T. Kleine, Astronomical context of Solar System formation from molybdenum isotopes in meteorite inclusions. Science 370, 837–840 (2020). 33184211 10.1126/science.aaz8482

[56] K. Metzler, D. C. Hezel, J. Barosch, E. Wölfer, J. M. Schneider, J. L. Hellmann, J. Berndt, A. Stracke, J. Gattacceca, R. C. Greenwood, I. A. Franchi, C. Burkhardt, T. Kleine, The Loongana (CL) group of carbonaceous chondrites. Geochim. Cosmochim. Acta 304, 1–31 (2021).

[57] K. Zhu, F. Moynier, M. Schiller, C. M. . O.’. D. Alexander, J. Davidson, D. L. Schrader, E. van Kooten, M. Bizzarro, Chromium isotopic insights into the origin of chondrite parent bodies and the early terrestrial volatile depletion. Geochim. Cosmochim. Acta 301, 158–186 (2021). 34393262 10.1016/j.gca.2021.02.031 PMC7611480

[58] D. L. Cook, I. Leya, M. Schönbächler, Galactic cosmic ray effects on iron and nickel isotopes in iron meteorites. Meteorit. Planet. Sci. 55, 2758–2771 (2020). 33664561 10.1111/maps.13446 PMC7891426

[59] B. M. Elfers, P. Sprung, N. Messling, C. Münker, The combined Zr and Hf isotope inventory of bulk rock and sequentially leached chondrite samples. Geochim. Cosmochim. Acta 270, 475–491 (2020).

[60] C. D. Hilton, K. R. Bermingham, R. J. Walker, T. J. McCoy, Genetics, crystallization sequence, and age of the South Byron Trio iron meteorites: New insights to carbonaceous chondrite (CC) type parent bodies. Geochim. Cosmochim. Acta 251, 217–228 (2019). 33273745 10.1016/j.gca.2019.02.035 PMC7709739

[61] N. S. Saji, D. Wielandt, J. C. Holst, M. Bizzarro, Solar system Nd isotope heterogeneity: Insights into nucleosynthetic components and protoplanetary disk evolution. Geochim. Cosmochim. Acta 281, 135–148 (2020).

[62] P. Frossard, Z. Guo, M. Spencer, M. Boyet, A. Bouvier, Evidence from achondrites for a temporal change in Nd nucleosynthetic anomalies within the first 1.5 million years of the inner Solar System formation. Earth Planet. Sci. Lett. 566, 116968 (2021).

