# Supplementary material for: Terrestrial planet formation from lost inner solar system material
Source: Sci Adv. 2021 Dec 22;7(52):eabj7601. doi: 10.1126/sciadv.abj7601 (PMC8694615; doi:10.1126/sciadv.abj7601)
Supplement: Supplementary file 1 — Figs S1 to S3 Tables S1 to S5 Legend for data S1 References [file sciadv.abj7601_sm.pdf]

Science Advances
AAAS

Supplementary Materials for

Terrestrial planet formation from lost inner solar system material

Christoph Burkhardt*, Fridolin Spitzer, Alessandro Morbidelli, Gerrit Budde, Jan H. Render, Thomas S. Kruijer, Thorsten Kleine

*Corresponding author. Email: burkhardt@uni-muenster.de

Published 22 December 2021, *Sci. Adv.* **7**, eabj7601 (2021)
DOI: 10.1126/sciadv.abj7601

The PDF file includes:

Figs S1 to S3
Tables S1 to S5
Legend for data S1
References

Other Supplementary Material for this manuscript includes the following:

Data S1

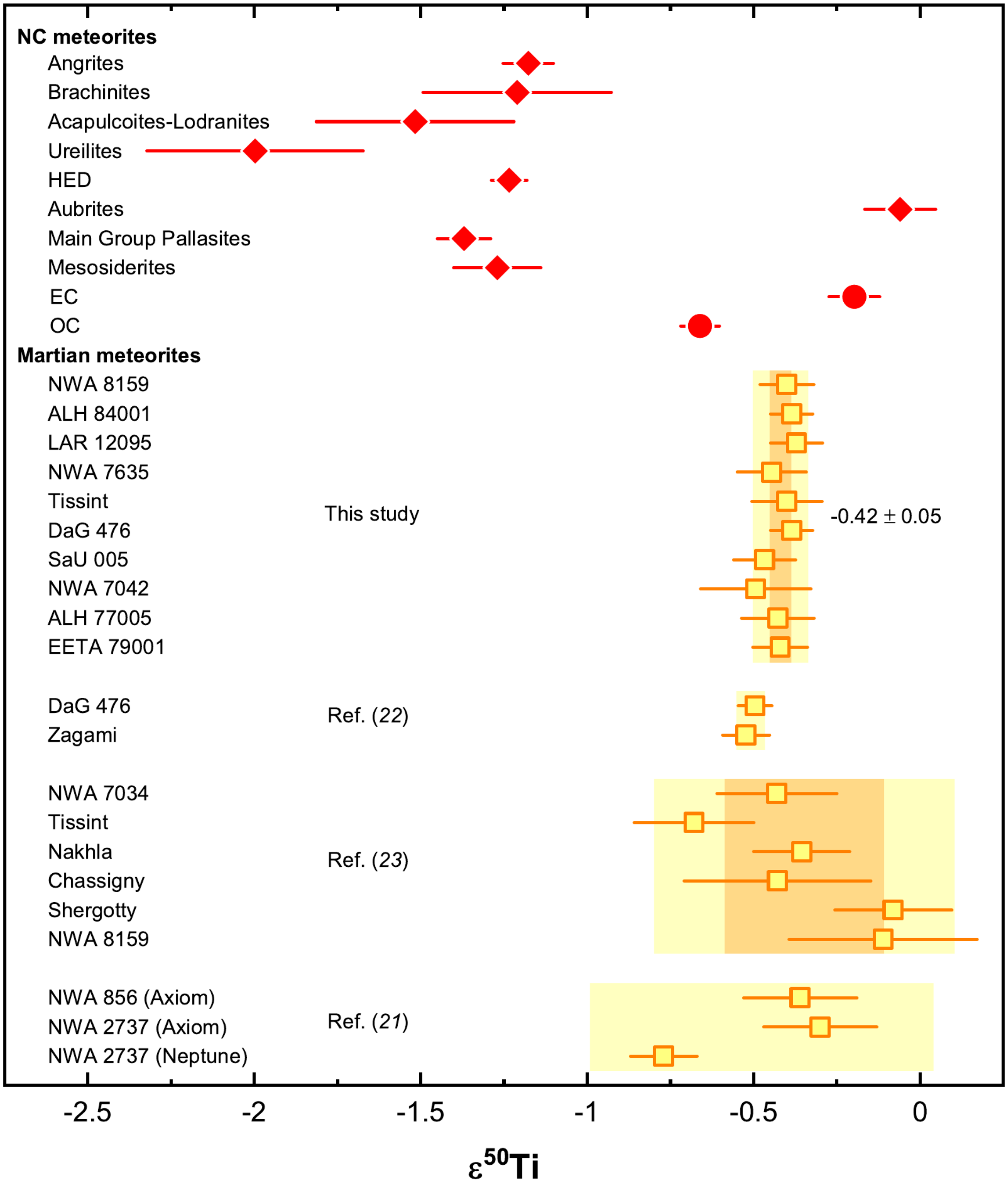

Fig. S1. Isotope anomalies in ε^{50}Ti for individual martian meteorites obtained here in comparison to literature data of martian meteorites and average NC bodies. Data and data sources for NC meteorites are given in the Supplementary Materials Data S1. Individual error bars and orange band represent two-sided Student's t-values 95% confidence intervals, yellow band two times the standard deviation.

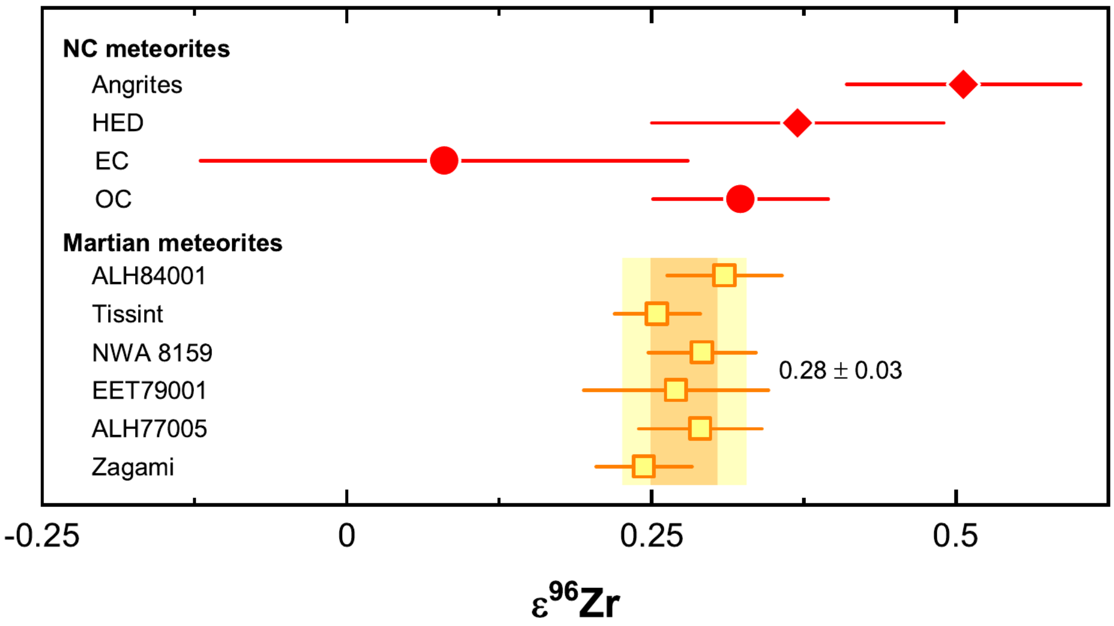

Fig. S2. Anomalies in ε^{96}Zr for individual martian meteorites obtained here in comparison to literature data of NC bodies. Data and data sources for NC meteorites are given in the Supplementary Materials Data S1. Individual error bars and orange band represent two-sided Student's t-values 95% confidence intervals, yellow band two times the standard deviation.

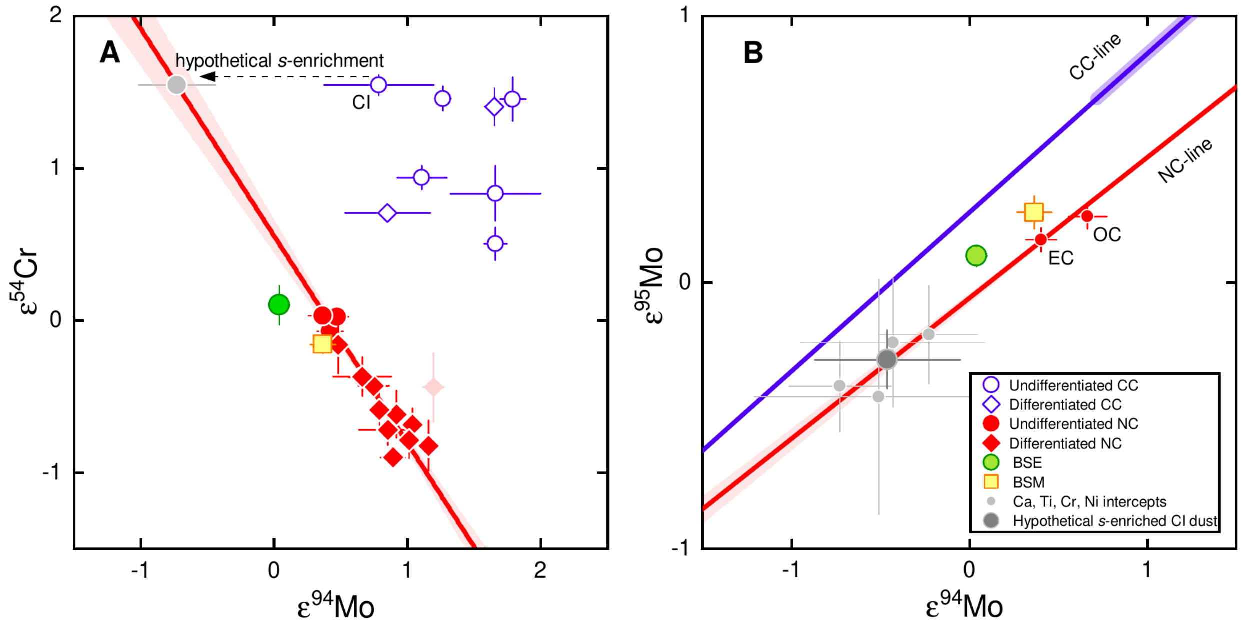

Fig. S3. Position of putative *s*-process enriched CC dust in ε^{54}Cr-ε^{94}Mo and ε^{95}Mo-ε^{94}Mo isotope space. (**A**) Extrapolation of the NC mixing trend to the intersection with the Ca, Ti, Cr, and Ni isotopic composition of CI meteorites allow to quantify the isotopic composition of a putative *s*-process-enriched CC dust component. When this is done using the NC correlations of ε^{92}Mo, ε^{94}Mo, ε^{95}Mo, ε^{97}Mo, and ε^{100}Mo with ε^{48}Ca, ε^{50}Ti, ε^{54}Cr, and ε^{62}Ni, respectively, the following Mo isotopic compositions for the putative *s*-enriched CC dust component are obtained: For a CI-like ε^{48}Ca of 2.11±0.20: ε^{92}Mo= -0.41±0.33, ε^{94}Mo= -0.43±0.29, ε^{95}Mo= -0.23±0.13, ε^{97}Mo= -0.35±0.11, ε^{100}Mo= 0.12±0.98. For a CI-like ε^{50}Ti of 1.89±0.15: ε^{92}Mo= -0.31±0.18, ε^{94}Mo= -0.23±0.14, ε^{95}Mo= -0.20±0.10, ε^{97}Mo= -0.02±0.05, ε^{100}Mo= 0.07±0.07. For a CI-like ε^{54}Cr of 1.55±0.07: ε^{92}Mo= -0.89±0.36, ε^{94}Mo= -0.73±0.29, ε^{95}Mo= -0.39±0.17, ε^{97}Mo= -0.26±0.14, ε^{100}Mo= -0.10±0.19. For a CI-like ε^{62}Ni of 0.17±0.09: ε^{92}Mo= -0.43±0.55, ε^{94}Mo= -0.51±0.55, ε^{95}Mo= -0.^{43}±0.33, ε^{97}Mo= -0.25±0.19, ε^{100}Mo= -0.16±0.16. All of these compositions (small gray points in **B**) are similar to one another, resulting in a weighted mean *s*-process Mo excess of ε^{92}Mo= -0.56±0.49, ε^{94}Mo= -0.46±0.41, ε^{95}Mo= -0.29±0.11, ε^{97}Mo= -0.18±0.24, ε^{100}Mo= -0.10±0.13 (large gray point in **B**). However, all these compositions plot on the NC-line close to the BSE in ε^{95}Mo-ε^{94}Mo isotope space, and not on the intersection of the NC and CC line, as would be expected for an internally consistent model. Thus, the missing component to explain the NC mixing trends cannot be inward drifting *s*-process enriched CC dust. Using the average isotopic composition of the CC bodies instead of CI values in the extrapolation does not change this conclusion. Uncertainties represent 95% confidence intervals.

Table S1. Titanium isotopic composition of martian meteorites.

Sample	Type	N	$\varepsilon^{46}Ti \pm$ 95% ci	$\varepsilon^{48}Ti \pm$ 95% ci	$\varepsilon^{50}Ti \pm$ 95% ci
DaG476	Depleted shergottite	12	-0.13 ± 0.06	0.02 ± 0.05	-0.39 ± 0.06
LAR12095	Depleted shergottite	12	-0.12 ± 0.06	0.02 ± 0.12	-0.37 ± 0.08
NWA7635	Depleted shergottite	11	-0.14 ± 0.11	0.04 ± 0.08	-0.45 ± 0.10
SaU005	Depleted shergottite	12	-0.12 ± 0.13	-0.01 ± 0.06	-0.47 ± 0.09
Tissint	Depleted shergottite	7	-0.01 ± 0.19	0.03 ± 0.09	-0.40 ± 0.11
ALH77005	Intermediate shergottite	12	0.01 ± 0.08	0.06 ± 0.15	-0.43 ± 0.11
EETA79001	Intermediate shergottite	12	-0.16 ± 0.06	0.06 ± 0.04	-0.42 ± 0.08
NWA7042	Intermediate shergottite	7	-0.11 ± 0.13	-0.05 ± 0.11	-0.49 ± 0.17
ALH84001	Orthopyroxenite	11	-0.13 ± 0.06	0.02 ± 0.05	-0.39 ± 0.06
NWA8159	Augite basalt	7	-0.10 ± 0.09	0.03 ± 0.05	-0.40 ± 0.08
**Mars mean**		**10**	**-0.10 ± 0.07**	**0.02 ± 0.04**	**-0.42 ± 0.05**

N = number of measurements; $\varepsilon^{i}Ti = [({}^{i}Ti/{}^{47}Ti)_{sample}/({}^{i}Ti/{}^{47}Ti)_{standard} - 1] \times 10^4$, where all ratios have been corrected for mass fractionation by internal normalization to fixed $^{49}Ti/^{47}Ti$ using the exponential law. Uncertainties represent two-sided Student's t-values 95% confidence intervals.

Table S2. Zirconium isotopic composition of martian meteorites and terrestrial rock standards.

Sample	Type	N	$\varepsilon^{91}Zr$ ± 95% ci	$\varepsilon^{92}Zr$ ± 95% ci	$\varepsilon^{96}Zr$ ± 95% ci
Tissint	Depleted shergottite	8	-0.02 ± 0.03	-0.04 ± 0.02	0.25 ± 0.04
ALH77005	Intermediate shergottite	10	0.01 ± 0.01	-0.02 ± 0.02	0.29 ± 0.06
EET79001	Intermediate shergottite	9	0.01 ± 0.03	0.00 ± 0.01	0.27 ± 0.09
Zagami	Enriched shergottite	17	-0.01 ± 0.02	-0.01 ± 0.02	0.24 ± 0.04
NWA8159	Augite basalt	8	-0.02 ± 0.04	-0.04 ± 0.02	0.29 ± 0.05
ALH84001	Orthopyroxenite	13	0.00 ± 0.02	-0.01 ± 0.02	0.31 ± 0.05
**Mars mean**		**6**	**0.00 ± 0.01**	**-0.02 ± 0.02**	**0.28 ± 0.03**
BHVO-2	Hawaiian basalt standard	18	0.01 ± 0.02	0.00 ± 0.02	0.01 ± 0.07
BCR-2	Intraplate basalt standard	17	-0.01 ± 0.02	-0.01 ± 0.02	-0.04 ± 0.04
**terrestrial mean**		**35**	**0.00 ± 0.01**	**0.00 ± 0.01**	**-0.01 ± 0.04**

N= number of measurements; $\varepsilon^{i}Zr = [(^{i}Zr/^{90}Zr)_{sample}/(^{i}Zr/^{90}Zr)_{standard} - 1]\times10^4$, where all ratios have been corrected for mass fractionation by internal normalization to fixed $^{94}Zr/^{90}Zr$ using the exponential law. Uncertainties represent two-sided Student's t-values 95% confidence intervals. BCR-2 data were obtained as part of the same measurement campaign, and have been reported in ref. (*47*).

Table S3. Molybdenum isotopic composition of martian meteorites.

Sample	Type	N	ε^{92}Mo ± 2se	ε^{94}Mo ± 2se	ε^{95}Mo ± 2se	ε^{97}Mo ± 2se	ε^{100}Mo ± 2se
DaG476	Depleted shergottite	1	0.42 ± 0.22	0.34 ± 0.15	0.28 ± 0.12	0.16 ± 0.09	-0.01 ± 0.13
DaG476	Depleted shergottite	1	0.49 ± 0.21	0.33 ± 0.15	0.19 ± 0.12	0.14 ± 0.09	-0.06 ± 0.13
Tissint 1	Depleted shergottite	1	0.75 ± 0.21	0.62 ± 0.15	0.37 ± 0.11	0.14 ± 0.07	-0.01 ± 0.12
Tissint 2	Depleted shergottite	1	0.76 ± 0.22	0.42 ± 0.17	0.19 ± 0.12	0.07 ± 0.07	0.06 ± 0.15
A (SaU005; DaG476)	Depleted shergottites	1	0.49 ± 0.22	0.27 ± 0.16	0.19 ± 0.11	0.12 ± 0.08	0.08 ± 0.16
B (ALH77005; EETA79001; NWA7042)	Intermediate shergottites	1	0.55 ± 0.20	0.36 ± 0.14	0.34 ± 0.11	0.23 ± 0.09	0.08 ± 0.14
C (NWA8159; RBT04262; NWA4864; LAR12011; LAR12095; ALH84001; NWA7635; Zagami; Nakhla; NWA10153; MIL03346)	Enriched shergottites; intermediate shergottites; depleted shergottites; Nakhlites; orthopyroxenite; augite basalt	1	0.50 ± 0.23	0.24 ± 0.15	0.22 ± 0.12	0.16 ± 0.10	0.01 ± 0.15
Tissint 2 and A-C (combined remains)	All types	1	0.66 ± 0.22	0.35 ± 0.16	0.32 ± 0.11	0.13 ± 0.08	0.16 ± 0.13
**Martian mantle mean**		**8**	**0.58 ± 0.11**	**0.37 ± 0.10**	**0.26 ± 0.06**	**0.14 ± 0.04**	**0.04 ± 0.06**

N= number of measurements; $\varepsilon^{i}\text{Mo} = [(^{i}\text{Mo}/^{96}\text{Mo})_{\text{sample}}/(^{i}\text{Mo}/^{96}\text{Mo})_{\text{standard}} - 1] \times 10^4$, where all ratios have been corrected for mass fractionation by internal normalization to fixed ^{98}Mo/^{96}Mo using the exponential law. Due to low Mo content, some samples were combined to obtain enough Mo for a single measurement (samples A, B, C, and Tissint 2 and A-C combined remains). Uncertainties represent 2 s.e. for individual measurements and two-sided Student's t-values 95% confidence intervals for martian mantle average.

Table S4. Mean values and the 1σ uncertainty of all independent quantities considered in the Monte Carlo calculation for the Earth.

coefficient	Mean	σ
a_{95Mo}	0.528	0.0225
a_{54Cr}	-1.456	0.145
a_{96Zr}	1.2	0.485
a_{145Nd}	0.1	0.035
$\varepsilon^{94}Mo^{CC}$	1.65	0.855
$\varepsilon^{54}Cr^{CC}$	1.14	0.305
$\varepsilon^{96}Zr^{CC}$	0.77	0.275
$\varepsilon^{145}Nd^{CC}$	0.03	0.025
$\varepsilon^{94}Mo^{BSE}$	0.04	0.03
$\varepsilon^{95}Mo^{BSE}$	0.1	0.02
$\varepsilon^{54}Cr^{BSE}$	0.1	0.065
$\varepsilon^{96}Zr^{BSE}$	0.01	0.015
$\varepsilon^{145}Nd^{BSE}$	-0.01	0.015

Table S5. Mean values and the 1σ uncertainty of independent quantities considered in the Monte Carlo calculation for Mars.

coefficient	Mean	σ
a_{95Mo}	0.528	0.0225
a_{54Cr}	-1.456	0.145
a_{96Zr}	1.2	0.485
$\varepsilon^{94}Mo^{CC}$	1.65	0.855
$\varepsilon^{54}Cr^{CC}$	1.14	0.305
$\varepsilon^{96}Zr^{CC}$	0.77	0.275
$\varepsilon^{94}Mo^{BSM}$	0.33	0.045
$\varepsilon^{95}Mo^{BSM}$	0.25	0.045
$\varepsilon^{54}Cr^{BSM}$	-0.16	0.015
$\varepsilon^{96}Zr^{BSM}$	0.28	0.01

Data S1. (separate file)

Data file S1 contains the compiled nucleosynthetic anomaly data that were used to prepare the Figures.

REFERENCES AND NOTES

1. J. E. Chambers, G. W. Wetherill, Making the terrestrial planets: N-body integrations of planetary embryos in three dimensions. *Icarus* **136**, 304–327 (1998).

2. M. Lambrechts, A. Johansen, Rapid growth of gas-giant cores by pebble accretion. *Astron. Astrophys.* **544**, A32 (2012).

3. C. W. Ormel, H. H. Klahr, The effect of gas drag on the growth of protoplanets. Analytical expressions for the accretion of small bodies in laminar disks. *Astron. Astrophys.* **520**, A43 (2010).

4. M. Lambrechts, A. Johansen, Forming the cores of giant planets from the radial pebble flux in protoplanetary discs. *Astron. Astrophys.* **572**, A107 (2014).

5. H. F. Levison, K. A. Kretke, M. J. Duncan, Growing the gas-giant planets by the gradual accumulation of pebbles. *Nature* **524**, 322–324 (2015).

6. H. F. Levison, K. A. Kretke, K. J. Walsh, W. F. Bottke, Growing the terrestrial planets from the gradual accumulation of submeter-sized objects. *Proc. Natl. Acad. Sci.* **112**, 14180–14185 (2015).

7. A. Johansen, T. Ronnet, M. Bizzarro, M. Schiller, M. Lambrechts, Å. Nordlund, H. Lammer, A pebble accretion model for the formation of the terrestrial planets in the Solar System. *Sci. Adv*. **7**, eabc0444 (2021).

8. S. N. Raymond, A. Izidoro, Origin of water in the inner Solar System: Planetesimals scattered inward during Jupiter and Saturn's rapid gas accretion. *Icarus* **297**, 134–148 (2017).

9. M. Schiller, M. Bizzarro, J. Siebert, Iron isotope evidence for very rapid accretion and differentiation of the proto-Earth. *Sci. Adv*. **6**, eaay7604 (2020).

10. M. Schiller, M. Bizzarro, V. A. Fernandes, Isotopic evolution of the protoplanetary disk and the building blocks of Earth and the Moon. *Nature* **555**, 507 (2018), 510.

11. P. H. Warren, Stable-isotopic anomalies and the accretionary assemblage of the Earth and Mars: A subordinate role for carbonaceous chondrites. *Earth Planet. Sci. Lett.* **311**, 93–100 (2011).

12. C. Burkhardt, T. Kleine, F. Oberli, A. Pack, B. Bourdon, R. Wieler, Molybdenum isotope anomalies in meteorites: Constraints on solar nebula evolution and origin of the Earth. *Earth Planet. Sci. Lett.* **312**, 390–400 (2011).

13. N. Dauphas, The isotopic nature of the Earth's accreting material through time. *Nature* **541**, 521–524 (2017).

14. K. R. Bermingham, E. A. Worsham, R. J. Walker, New insights into Mo and Ru isotope variation in the nebula and terrestrial planet accretionary genetics. *Earth Planet. Sci. Lett.* **487**, 221–229 (2018).

15. R. Brasser, N. Dauphas, S. J. Mojzsis, Jupiter's influence on the building blocks of Mars and Earth. *Geophys. Res. Lett.,* **45**, 5908–5917 (2018).

16. H. Tang, N. Dauphas, ^{60}Fe–^{60}Ni chronology of core formation in Mars. *Earth Planet. Sci. Lett.* **390**, 264–274 (2014).

17. K. Mezger, M. Schönbächler, A. Bouvier, Accretion of the Earth—Missing components? *Space Sci. Rev*. **216**, 27 (2020).

18. G. Budde, C. Burkhardt, T. Kleine, Molybdenum isotopic evidence for the late accretion of outer solar system material to Earth. *Nat. Astron.* **3**, 736–741 (2019).

19. M. Fischer-Gödde, T. Kleine, Ruthenium isotopic evidence for an inner solar system origin of the late veneer. *Nature* **541**, 525–527 (2017).

20. F. Spitzer, C. Burkhardt, G. Budde, T. S. Kruijer, A. Morbidelli, T. Kleine, Isotopic evolution of the inner solar system inferred from molybdenum isotopes in meteorites. *Astrophys. J.* **898**, L2 (2020).

21. A. Trinquier, T. Elliott, D. Ulfbeck, C. Coath, A. N. Krot, M. Bizzarro, Origin of nucleosynthetic isotope heterogeneity in the solar protoplanetary disk. *Science* **324**, 374–376 (2009).

22. C. Burkhardt, N. Dauphas, H. Tang, M. Fischer-Gödde, L. Qin, J. H. Chen, S. S. Rout, A. Pack, P. R. Heck, D. A. Papanastassiou, In search of the Earth-forming reservoir: Mineralogical, chemical, and isotopic characterizations of the ungrouped achondrite NWA 5363/NWA 5400 and selected chondrites. *Meteorit. Planet. Sci.* **52**, 807–826 (2017).

23. C. D. Williams, M. E. Sanborn, C. Defouilloy, QZ. . Yin, N. T. Kita, D. S. Ebel, A. Yamakawa, K. Yamashita, Chondrules reveal large-scale outward transport of inner solar system materials in the protoplanetary disk. *Proc. Natl. Acad. Sci. U.S.A.* **117**, 23426–23435 (2020).

24. C. Burkhardt, L. E. Borg, G. A. Brennecka, Q. R. Shollenberger, N. Dauphas, T. Kleine, A nucleosynthetic origin for the Earth's anomalous ^{142}Nd composition. *Nature* **537**, 394–398 (2016).

25. T. Kleine, G. Budde, C. Burkhardt, T. S. Kruijer, E. A. Worsham, A. Morbidelli, F. Nimmo, The noncarbonaceous-carbonaceous meteorite dichotomy. *Space Sci. Rev*. **216**, 55 (2020).

26. T. Haugbølle, P. Weber, D. P. Wielandt, P. Benítez-Llambay, M. Bizzarro, O. Gressel, M. E. Pessah, Probing the protosolar disk using dust filtering at gaps in the early solar system. *Astron. J.* **158**, 55 (2019).

27. P. Weber, P. Benítez-Llambay, O. Gressel, L. Krapp, M. E. Pessah, Characterizing the variable dust permeability of planet-induced gaps. *Astrophys. J.* **854**, 153 (2018).

28. M. Ek, A. C. Hunt, M. Lugaro, M. Schönbächler, The origin of *s*-process isotope heterogeneity in the solar protoplanetary disk. *Nat. Astron.* **4**, 273–281 (2020).

29. S. J. Desch, A. Kalyaan, C. M. O'D. Alexander, The effect of Jupiter's formation on the distribution of refractory elements and inclusions in meteorites. *Astrophys. J. Suppl. Ser.* **238**, 11 (2018).

30. T. S. Kruijer, C. Burkhardt, G. Budde, T. Kleine, Age of Jupiter inferred from the distinct genetics and formation times of meteorites. *Proc. Natl. Acad. Sci. U.S.A.* **114**, 6712–6716 (2017).

31. K. J. Walsh, A. Morbidelli, S. N. Raymond, D. P. O'Brien, A. M. Mandell, A low mass for Mars from Jupiter's early gas-driven migration. *Nature* **475**, 206–209 (2011).

32. R. Brasser, S. J. Mojzsis, The partitioning of the inner and outer Solar System by a structured protoplanetary disk. *Nat. Astron.* **4**, 492–499 (2020).

33. S. Charnoz, G. Avice, R. Hyodo, F. C. Pignatale, M. Chaussidon, Forming pressure traps at the snow line to isolate isotopic reservoirs in the absence of a planet. *A&A* **652**, A35 (2021).

34. T. Lichtenberg, J. Drążkowska, M. Schönbächler, G. J. Golabek, T. O. Hands, Bifurcation of planetary building blocks during Solar System formation. *Science* **371**, 365–370 (2021).

35. M. Lambrechts, A. Johansen, A. Morbidelli, Separating gas-giant and ice-giant planets by halting pebble accretion. *Astron. Astrophys.* **572**, A35 (2014).

36. N. Dauphas, A. Pourmand, Hf-W-Th evidence for rapid growth of Mars and its status as a planetary embryo. *Nature* **473**, 489–492 (2011).

37. D. C. Rubie, S. A. Jacobson, A. Morbidelli, D. P. O'Brien, E. D. Young, J. de Vries, F. Nimmo, H. Palme, D. J. Frost, Accretion and differentiation of the terrestrial planets with implications for the compositions of early-formed Solar System bodies and accretion of water. *Icarus* **248**, 89–108 (2015).

38. B. Marty, The origins and concentrations of water, carbon, nitrogen and noble gases on Earth. *Earth Planet. Sci. Lett.* **313-314**, 56–66 (2012).

39. L. Piani, Y. Marrocchi, T. Rigaudier, L. G. Vacher, D. Thomassin, B. Marty, Earth's water may have been inherited from material similar to enstatite chondrite meteorites. *Science* **369**, 1110–1113 (2020).

40. K. Lodders, B. Fegley, An oxygen isotope model for the composition of Mars. *Icarus* **126**, 373–394 (1997).

41. D. P. O'Brien, K. J. Walsh, A. Morbidelli, S. N. Raymond, A. M. Mandell, Water delivery and giant impacts in the 'Grand Tack' scenario. *Icarus* **239**, 74–84 (2014).

42. T. S. Kruijer, L. E. Borg, J. Wimpenny, C. K. Sio, Onset of magma ocean solidification on Mars inferred from Mn-Cr chronometry. *Earth Planet. Sci. Lett.* **542**, 116315 (2020).

43. T. S. Kruijer, T. Kleine, L. E. Borg, G. A. Brennecka, A. J. Irving, A. Bischoff, C. B. Agee, The early differentiation of Mars inferred from Hf-W chronometry. *Earth Planet. Sci. Lett.* **474**, 345–354 (2017).

44. L. E. Borg, G. A. Brennecka, S. J. K. Symes, Accretion timescale and impact history of Mars deduced from the isotopic systematics of martian meteorites. *Geochim. Cosmochim. Acta* **175**, 150–167 (2016).

45. S. Gerber, C. Burkhardt, G. Budde, K. Metzler, T. Kleine, Mixing and transport of dust in the early solar nebula as inferred from titanium isotope variations among chondrules. *Astrophys. J. Lett.* **841**, L17 (2017).

46. T. S. Kruijer, T. Kleine, M. Fischer-Gödde, P. Sprung, Lunar tungsten isotopic evidence for the late veneer. *Nature* **520**, 534–537 (2015).

47. J. Render, G. A. Brennecka, Isotopic signatures as tools to reconstruct the primordial architecture of the Solar System. *Earth Planet. Sci. Lett.* **555**, 116705 (2021).

48. J. J. Zhang, N. Dauphas, A. M. Davis, A. Pourmand, A new method for MC-ICPMS measurement of titanium isotopic composition: Identification of correlated isotope anomalies in meteorites. *J. Anal. At. Spectrom.* **26**, 2197–2205 (2011).

49. J. J. Zhang, N. Dauphas, A. M. Davis, I. Leya, A. Fedkin, The proto-Earth as a significant source of lunar material. *Nat. Geosci.* **5**, 251–255 (2012).

50. R. Bast, E. E. Scherer, P. Sprung, K. Mezger, M. Fischer-Gödde, S. Taetz, M. Böhnke, H. Schmid-Beurmann, C. Münker, T. Kleine, G. Srinivasan, Reconciliation of the excess ^{176}Hf conundrum in meteorites: Recent disturbances of the Lu-Hf and Sm-Nd isotope systematics. *Geochim. Cosmochim. Acta* **212**, 303–323 (2017).

51. G. Budde, C. Burkhardt, G. A. Brennecka, M. Fischer-Gödde, T. S. Kruijer, T. Kleine, Molybdenum isotopic evidence for the origin of chondrules and a distinct genetic heritage of carbonaceous and non-carbonaceous meteorites. *Earth Planet. Sci. Lett.* **454**, 293–303 (2016).

52. T. Hopp, G. Budde, T. Kleine, Heterogeneous accretion of Earth inferred from Mo-Ru isotope systematics. *Earth Planet. Sci. Lett.* **534**, 116065 (2020).

53. C. Burkhardt, N. Dauphas, U. Hans, B. Bourdon, T. Kleine, Elemental and isotopic variability in solar system materials by mixing and processing of primordial disk reservoirs. *Geochim. Cosmochim. Acta* **261**, 145–170 (2019).

54. Z. A. Torrano, D. L. Schrader, J. Davidson, R. C. Greenwood, D. R. Dunlap, M. Wadhwa, The relationship between CM and CO chondrites: Insights from combined analyses of titanium, chromium, and oxygen isotopes in CM, CO, and ungrouped chondrites. *Geochim. Cosmochim. Acta* **301**, 70–90 (2021).

55. G. A. Brennecka, C. Burkhardt, G. Budde, T. S. Kruijer, F. Nimmo, T. Kleine, Astronomical context of Solar System formation from molybdenum isotopes in meteorite inclusions. *Science* **370**, 837–840 (2020).

56. K. Metzler, D. C. Hezel, J. Barosch, E. Wölfer, J. M. Schneider, J. L. Hellmann, J. Berndt, A. Stracke, J. Gattacceca, R. C. Greenwood, I. A. Franchi, C. Burkhardt, T. Kleine, The Loongana (CL) group of carbonaceous chondrites. *Geochim. Cosmochim. Acta* **304**, 1–31 (2021).

57. K. Zhu, F. Moynier, M. Schiller, CM. .O.'D. Alexander, J. Davidson, D. L. Schrader, E. van Kooten, M. Bizzarro, Chromium isotopic insights into the origin of chondrite parent bodies and the early terrestrial volatile depletion. *Geochim. Cosmochim. Acta* **301**, 158–186 (2021).

58. D. L. Cook, I. Leya, M. Schönbächler, Galactic cosmic ray effects on iron and nickel isotopes in iron meteorites. *Meteorit. Planet. Sci.* **55**, 2758–2771 (2020).

59. B. M. Elfers, P. Sprung, N. Messling, C. Münker, The combined Zr and Hf isotope inventory of bulk rock and sequentially leached chondrite samples. *Geochim. Cosmochim. Acta* **270**, 475–491 (2020).

60. C. D. Hilton, K. R. Bermingham, R. J. Walker, T. J. McCoy, Genetics, crystallization sequence, and age of the South Byron Trio iron meteorites: New insights to carbonaceous chondrite (CC) type parent bodies. *Geochim. Cosmochim. Acta* **251**, 217–228 (2019).

61. N. S. Saji, D. Wielandt, J. C. Holst, M. Bizzarro, Solar system Nd isotope heterogeneity: Insights into nucleosynthetic components and protoplanetary disk evolution. *Geochim. Cosmochim. Acta* **281**, 135–148 (2020).

62. P. Frossard, Z. Guo, M. Spencer, M. Boyet, A. Bouvier, Evidence from achondrites for a temporal change in Nd nucleosynthetic anomalies within the first 1.5 million years of the inner Solar System formation. *Earth Planet. Sci. Lett.* **566**, 116968 (2021).
