# Supplementary material for: Terrestrial planet formation from lost inner solar system material
Source: Sci Adv. 2021 Dec 22;7(52):eabj7601. doi: 10.1126/sciadv.abj7601 (PMC8694615; doi:10.1126/sciadv.abj7601)
Supplement: Supplementary file 2 — Data S1 [file sciadv.abj7601_data_file_s1.zip › sciadv.abj7601_data_file_s1.pdf]

Table S1. Nucleosynthetic anomalies in planetary materials.

	Reservoir	ε^{48}Ca ± 95% CI	ε^{50}Ti ± 95% CI	ε^{54}Cr ± 95% CI	ε^{54}Fe ± 95% CI	ε^{62}Ni ± 95% CI	ε^{96}Zr ± 95% CI	ε^{94}Mo ± 95% CI	ε^{95}Mo ± 95% CI	ε^{100}Ru ± 95% CI	ε^{145}Nd ± 95% CI
Carbonaceous Chondrites											
CI	CC	2.11 ± 0.20	1.89 ± 0.15	1.55 ± 0.07	-0.02 ± 0.03	0.17 ± 0.09	0.34 ± 0.54	0.79 ± 0.41	0.69 ± 0.23	-0.24 ± 0.13	0.02 ± 0.02
CM	CC	3.14 ± 0.14	2.89 ± 0.15	0.97 ± 0.08	0.23 ± 0.04	0.11 ± 0.03	0.70 ± 0.67	4.82 ± 0.20	3.17 ± 0.16	-0.69 ± 0.38	0.06 ± 0.03
CO	CC	3.87 ± 0.56	3.58 ± 0.85	0.83 ± 0.18	0.13 ± 0.08	0.11 ± 0.04	0.94 ± 0.20	1.66 ± 0.34	1.39 ± 0.34	-0.92 ± 0.98	0.06 ± 0.02
CV	CC	3.50 ± 0.38	3.45 ± 0.19	0.94 ± 0.08	0.22 ± 0.04	0.11 ± 0.03	0.96 ± 0.37	1.11 ± 0.19	0.93 ± 0.14	-1.17 ± 0.22	0.04 ± 0.04
CK	CC		3.42 ± 1.05	0.50 ± 0.11	0.26 ± 0.06		0.45 ± 0.25	1.66 ± 0.09	1.30 ± 0.17	-1.10 ± 0.23	
CR	CC	2.15 ± 0.22	2.51 ± 0.45	1.27 ± 0.06	0.29 ± 0.04	0.07 ± 0.08	1.03 ± 0.86	3.11 ± 0.15	2.26 ± 0.04	-0.76 ± 0.36	0.04 ± 0.04
CH	CC			1.45 ± 0.14	0.16 ± 0.07			1.79 ± 0.10	1.29 ± 0.04	-0.91 ± 0.13	
CB	CC		2.04 ± 0.07	1.35 ± 0.20		0.16 ± 0.11	0.96 ± 0.25	1.26 ± 0.04	0.99 ± 0.04	-1.04 ± 0.04	
CL	CC		2.60 ± 0.22	0.72 ± 0.08							
ungrouped											
Tagish Lake	CC	2.91 ± 0.05	2.76 ± 0.26	1.33 ± 0.26						-1.03 ± 0.13	-0.01 ± 0.04
NWA 1839	CC		3.20 ± 0.51	1.03 ± 0.07							
Flensburg	CC		2.98 ± 0.10	1.06 ± 0.11							
EET 83226	CC		4.25 ± 0.15	0.93 ± 0.13							
EET 83355	CC		3.11 ± 0.15	0.76 ± 0.13							
MAC 87300	CC		4.67 ± 0.15	0.71 ± 0.14							
NWA 5958	CC		3.34 ± 0.15	1.18 ± 0.16							
LEW 85332	CC		2.42 ± 0.15	1.23 ± 0.13							
MAC 88107	CC		3.03 ± 0.15	1.11 ± 0.15							
MAC 87301	CC		4.12 ± 0.15	0.83 ± 0.14							
GRO 95566	CC		3.50 ± 0.15	0.92 ± 0.13							
LAP 04757	NC		-0.19 ± 0.15	-0.33 ± 0.13							
LAP 04773	NC		-0.54 ± 0.15	-0.46 ± 0.16							
Ordinary Chondrites											
H	NC	-0.23 ± 0.03	-0.65 ± 0.14	-0.31 ± 0.13	0.08 ± 0.03	-0.06 ± 0.03	0.32 ± 0.08	0.72 ± 0.20	0.29 ± 0.05	-0.27 ± 0.04	0.07 ± 0.04
L	NC	-0.32 ± 0.11	-0.67 ± 0.10	-0.40 ± 0.06	0.09 ± 0.04	-0.04 ± 0.04	0.40 ± 0.40	0.60 ± 0.19	0.21 ± 0.03	-0.28 ± 0.13	0.04 ± 0.06
LL	NC	-0.44 ± 0.09	-0.67 ± 0.08	-0.42 ± 0.08	0.13 ± 0.03	-0.07 ± 0.03	0.34 ± 0.25	0.52 ± 0.10	0.18 ± 0.05	-0.05 ± 0.13	0.01 ± 0.03
OC Mean	NC	-0.31 ± 0.09	-0.66 ± 0.06	-0.37 ± 0.06	0.11 ± 0.03	-0.06 ± 0.02	0.32 ± 0.04	0.67 ± 0.11	0.25 ± 0.05	-0.24 ± 0.06	0.05 ± 0.03
Rumuruti chondrites											
R	NC			-0.07 ± 0.03	0.06 ± 0.01			0.42 ± 0.10	0.18 ± 0.05	-0.39 ± 0.13	
Enstatite chondrites											
EH	NC	-0.32 ± 0.56	-0.14 ± 0.07	0.02 ± 0.05		0.03 ± 0.03	0.04 ± 0.24	0.47 ± 0.09	0.18 ± 0.07	-0.08 ± 0.04	0.03 ± 0.04
EL	NC	-0.40 ± 0.51	-0.28 ± 0.17	0.03 ± 0.05	0.06 ± 0.01	-0.03 ± 0.07	0.20 ± 0.40	0.36 ± 0.11	0.14 ± 0.06	-0.08 ± 0.05	0.03 ± 0.02
EC Mean	NC	-0.37 ± 0.37	-0.20 ± 0.08	0.03 ± 0.03	0.06 ± 0.01	0.00 ± 0.03	0.08 ± 0.20	0.38 ± 0.08	0.15 ± 0.04	-0.08 ± 0.03	0.03 ± 0.02
Achondrites											
Acapulcoites-Lodranites	NC		-1.52 ± 0.30	-0.62 ± 0.15				0.92 ± 0.07	0.48 ± 0.03	-0.35 ± 0.05	
Brachinites	NC		-1.21 ± 0.28	-0.44 ± 0.23				1.20 ± 0.08	0.58 ± 0.05	0.25 ± 0.07	
Winonaites	NC	-0.21 ± 0.09						0.25 ± 0.15	0.09 ± 0.09	-0.06 ± 0.10	
Angrites	NC	-1.06 ± 0.33	-1.18 ± 0.08	-0.43 ± 0.06		0.01 ± 0.05	0.51 ± 0.10	0.75 ± 0.11	0.39 ± 0.06		0.07 ± 0.02
Aubrites	NC	-0.44 ± 0.59	-0.06 ± 0.11	-0.16 ± 0.19		0.05 ± 0.19		0.48 ± 0.05	0.25 ± 0.06	-0.06 ± 0.03	0.07 ± 0.07
HED	NC	-1.24 ± 0.29	-1.23 ± 0.05	-0.69 ± 0.08	0.12 ± 0.02	0.03 ± 0.12	0.37 ± 0.12				0.07 ± 0.01
Ureilites	NC	-1.46 ± 0.20	-2.00 ± 0.32	-0.90 ± 0.04	0.14 ± 0.03	-0.05 ± 0.16		0.89 ± 0.09	0.38 ± 0.04	-0.27 ± 0.11	
ungrouped											
NWA 5363/5400	NC	-0.53 ± 0.20	-1.02 ± 0.10	-0.37 ± 0.13		0.01 ± 0.03		0.66 ± 0.22	0.31 ± 0.15	-0.34 ± 0.13	0.11 ± 0.06
NWA 2526	NC							0.60 ± 0.13	0.39 ± 0.13	-0.08 ± 0.13	
NWA 6112	NC							1.55 ± 0.22	0.79 ± 0.15	-0.46 ± 0.07	
NWA 1058	NC							1.31 ± 0.11	0.68 ± 0.09	-0.40 ± 0.07	
NWA 8548	NC							1.53 ± 0.10	1.27 ± 0.07	-1.14 ± 0.10	
NWA 6926	NC							1.48 ± 0.12	1.14 ± 0.07	-1.04 ± 0.14	
NWA 7325	NC		-1.58 ± 0.33	-0.61 ± 0.11							
NWA 468	NC		-1.54 ± 0.42	-0.59 ± 0.09							
NWA 8054	NC		-1.01 ± 0.38	-0.44 ± 0.08							
GRV 020043	NC		-1.59 ± 0.24	-0.48 ± 0.10							
GRA 06128	NC		-1.44 ± 0.24	-0.43 ± 0.11							
GRA 06129	NC		-1.55 ± 0.27	-0.46 ± 0.13							
Bunburra Rockhole	NC			-0.36 ± 0.10			0.13 ± 0.03				0.08 ± 0.04
Tafassasset	CC		2.05 ± 0.10	1.40 ± 0.12				1.65 ± 0.07	1.20 ± 0.05	-1.15 ± 0.04	0.11 ± 0.02
NWA 8548	CC							1.53 ± 0.10	1.27 ± 0.07	-1.14 ± 0.10	
NWA 6926	CC							1.48 ± 0.12	1.14 ± 0.07	-1.04 ± 0.14	
NWA 3100	CC		1.91 ± 0.31	1.50 ± 0.11							
NWA 2788	CC		2.13 ± 0.51	1.04 ± 0.12							
NWA 7822	CC		2.13 ± 0.51	1.14 ± 0.08							
NWA 2994	CC		2.48 ± 0.96	1.31 ± 0.10							
NWA 6704	CC		2.07 ± 0.14	1.56 ± 0.10							0.09 ± 0.02
Mesosiderites	NC		-1.27 ± 0.13	-0.69 ± 0.11				1.04 ± 0.08	0.46 ± 0.05	-0.42 ± 0.02	
Pallasites											
Eagle station pallasites	CC			0.71 ± 0.01	0.27 ± 0.42			0.85 ± 0.32	0.80 ± 0.14		
Main group pallasites	NC		-1.37 ± 0.08	-0.72 ± 0.10	0.09 ± 0.09	-0.06 ± 0.10		0.85 ± 0.22	0.38 ± 0.14	-0.45 ± 0.23	
ungrouped											
Milton	CC			1.07 ± 0.07				1.30 ± 0.26	1.04 ± 0.09	-1.14 ± 0.15	
Iron meteorites											
IAB	NC				0.00 ± 0.06	-0.05 ± 0.06		0.04 ± 0.10	-0.07 ± 0.05	0.02 ± 0.09	
IC	NC				0.06 ± 0.05	-0.07 ± 0.04		0.90 ± 0.06	0.40 ± 0.03	-0.41 ± 0.05	
IIAB	NC			-0.82 ± 0.17	0.12 ± 0.06	-0.10 ± 0.07		1.16 ± 0.04	0.53 ± 0.03	-0.44 ± 0.05	
IIC	CC				0.32 ± 0.03	0.16 ± 0.08		2.31 ± 0.10	1.58 ± 0.05	-1.03 ± 0.04	
IID	CC					0.19 ± 0.06		1.18 ± 0.07	1.01 ± 0.03	-0.86 ± 0.27	
IIE	NC			-0.59 ± 0.13				0.79 ± 0.05	0.36 ± 0.03		0.04 ± 0.02
IIF	CC					0.09 ± 0.04		1.10 ± 0.03	0.95 ± 0.04	-1.02 ± 0.07	
IIIAB	NC			-0.79 ± 0.12	0.10 ± 0.00	-0.12 ± 0.02		1.01 ± 0.04	0.46 ± 0.04	-0.60 ± 0.06	
IIIE	NC					-0.07 ± 0.04		0.96 ± 0.02	0.46 ± 0.06	-0.55 ± 0.06	
IIIF	CC					0.12 ± 0.08		1.21 ± 0.05	0.97 ± 0.09	-1.02 ± 0.11	
IVA	NC					-0.07 ± 0.04		0.79 ± 0.10	0.36 ± 0.05	-0.29 ± 0.05	
IVB	CC				0.29 ± 0.07	0.07 ± 0.04		1.54 ± 0.10	1.16 ± 0.05	-0.90 ± 0.05	
ungrouped											
Wiley (IIC anomalous)	CC					0.13 ± 0.05		3.45 ± 0.10	2.24 ± 0.06	-1.09 ± 0.08	
South Byron Trio	CC							1.27 ± 0.07	1.03 ± 0.04	-1.07 ± 0.05	
Mbosi	CC							1.10 ± 0.43	1.02 ± 0.27		
Sombrerete (IAB complex)	CC							1.73 ± 0.29	1.15 ± 0.15		
Tishomingo	CC							1.42 ± 0.22	0.93 ± 0.13	-1.00 ± 0.14	
Chinga	CC							1.62 ± 0.08	1.13 ± 0.02	-1.04 ± 0.03	
Dronino	CC							1.39 ± 0.28	0.98 ± 0.15	-1.02 ± 0.15	
Mont Dieu	NC							0.63 ± 0.21	0.18 ± 0.13		
Gebel Kamil	NC							0.34 ± 0.30	0.07 ± 0.15	-0.03 ± 0.06	
Earth's mantle	BSE	0.00 ± 0.04	0.00 ± 0.00	0.10 ± 0.13	0.00 ± 0.02	0.03 ± 0.02	0.01 ± 0.03	0.04 ± 0.06	0.10 ± 0.04	0.02 ± 0.02	-0.01 ± 0.02
Mars' mantle	BSM	-0.20 ± 0.03	-0.42 ± 0.07	-0.16 ± 0.03	0.07 ± 0.02	0.04 ± 0.03	0.28 ± 0.03	0.37 ± 0.10	0.26 ± 0.06		0.02 ± 0.03
CAIs	IC	3.80 ± 1.33	8.57 ± 0.40	5.93 ± 0.52	0.41 ± 0.24	0.59 ± 0.17	1.76 ± 0.22	2.00 ± 1.55	2.31 ± 0.92	-1.60 ± 0.06	-0.22 ± 0.03

Uncertainties are Student-t 95% confidence intervals (CI), i.e. ($t_{0.95,\ N-1}$ x s.d.)/$\sqrt{N}$ for $N \geq 4$ and 2 s.d. for $N<4$. Data table is based on literature compilations (*20, 22, 53*), updated with recent publications (*9, 23, 42, 47, 52, 54–62*).
